# Capture and Culture of Circulating Tumor Cells Using a Biodegradable 3D Scaffold for Patient‐Derived Xenograft Construction

**DOI:** 10.1002/advs.202511148

**Published:** 2026-03-17

**Authors:** Yi‐Ke Wang, Ming Wang, Min Liu, Yi‐Jing Chen, Li‐Li Xu, Jin‐Ping Zhou, Fu‐Bing Wang, Shi‐Bo Cheng, Min Xie, Wei‐Hua Huang

**Affiliations:** ^1^ College of Chemistry and Molecular Sciences Wuhan University Wuhan P. R. China; ^2^ School of Laboratory Medicine, Hubei Shizhen Laboratory Hubei University of Chinese Medicine Wuhan P. R. China; ^3^ Center for Single‐Cell Omics and Tumor Liquid Biopsy Zhongnan Hospital of Wuhan University Wuhan P. R. China; ^4^ Henan Academy of Sciences Zhengzhou P. R. China; ^5^ Department of Clinical Laboratory Renmin Hospital of Wuhan University Wuhan P. R. China; ^6^ Department of Laboratory Medicine Zhongnan Hospital of Wuhan University Wuhan P. R. China

**Keywords:** biodegradable cellulose scaffold, circulating tumor cells, implantation, isolation and expansion, microfluidic chip

## Abstract

Patient‐derived xenograft (PDX) models constructed by noninvasively accessible circulating tumor cells (CTCs) serve as pivotal and invaluable tools for oncology research and precision medicine. However, the rarity of CTCs in blood and their limited proliferation ability present significant challenges for establishing patient CTC‐derived xenograft (CDX) models. In this study, a novel 3D cellulose scaffold is developed for efficient isolation and in situ culture of CTCs, with the aim of constructing CDX models. Characterized by a macroporous and flexible structure, the 3D cellulose scaffold achieves high‐efficiency CTC capture while maintaining cell viability. Its excellent biocompatibility and permeability further promote the rapid and in situ growth of CTCs. Importantly, the self‐supporting and biodegradable scaffold allows for direct implantation into immunodeficient mice without leaving any residual material. This 3D cellulose scaffold has been successfully employed to construct CDX models from breast cancer patients. The application of this platform may enhance our understanding of metastasis mechanisms and offer significant benefits for cancer therapy.

## Graphical abstract

A self‐supporting, biocompatible, and biodegradable 3D cellulose scaffold has been developed for efficient isolation and in situ expansion of circulating tumor cells (CTCs) from patient blood. Subsequently, the 3D cellulose scaffold with cultured CTCs is directly implanted into immunodeficient mice for constructing patient CTC‐derived xenograft (CDX) models, which replicate the invasive characteristics of a patient's tumor.

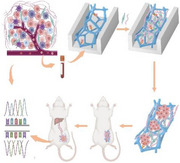

## 1 Introduction

Patient‐derived xenograft (PDX) models have been instrumental in oncology research due to their ability to closely mimic the tumor growth processes observed in patients [1]. These models reflect the molecular and histological characteristics of tumors, making them invaluable for cancer research, drug testing, and personalized treatment approaches [2, 3, 4]. However, the construction of PDX models requires invasive surgeries to obtain tumor tissues for implantation into mice. In some cases, tumor tissues may not be surgically accessible, limiting the broader clinical application of PDX models [2, 5, 6].

Circulating tumor cells (CTCs), on the other hand, offer a less invasive alternative [7]. These cells detach from the primary tumor, circulate in the bloodstream, and play a crucial role in metastasis [8]. CTCs retain the biological features of the primary tumor and have gained attention as key biomarkers in liquid biopsy for early cancer diagnosis, progression monitoring, and prognosis assessment [8, 9]. Given their noninvasive accessibility, CTCs hold great promise for the construction of patient CTC‐derived xenograft (CDX) models [10, 11, 12]. Additionally, CDX models, derived from highly invasive subpopulations of tumor cells, may better capture the molecular and metastatic characteristics of the patient's tumor, and can be constructed at various stages of cancer [13, 14]. This is invaluable for studying tumor progression mechanisms and improving clinical treatment outcomes.

However, constructing CDX models poses a challenge due to the rarity of CTCs in the bloodstream and their generally weak proliferative capacity [12, 15]. The harsh conditions of the circulatory system, immune surveillance, and mechanical forces during capture further hinder CTC viability [16, 17]. Efficient acquisition and expansion of CTCs are crucial for CDX construction, but current strategies are limited. One approach involves isolating and culturing CTCs in low‐adhesion plates under specific conditions, but this often damages cell viability and requires complex protocols [18, 19, 20, 21, 22, 23, 24, 25]. Alternatively, microfluidic chips can capture CTCs for in situ culture with stromal cells, but the obtained CTCs are confined in these chips and unsuitable for in vivo implantation to establish CDX, limiting downstream applications like intravital drug response test, cancer heterogeneity studies, etc [26, 27, 28, 29]. Therefore, there is an urgent need to develop an innovative technique for constructing CDX models.

**FIGURE 1:**
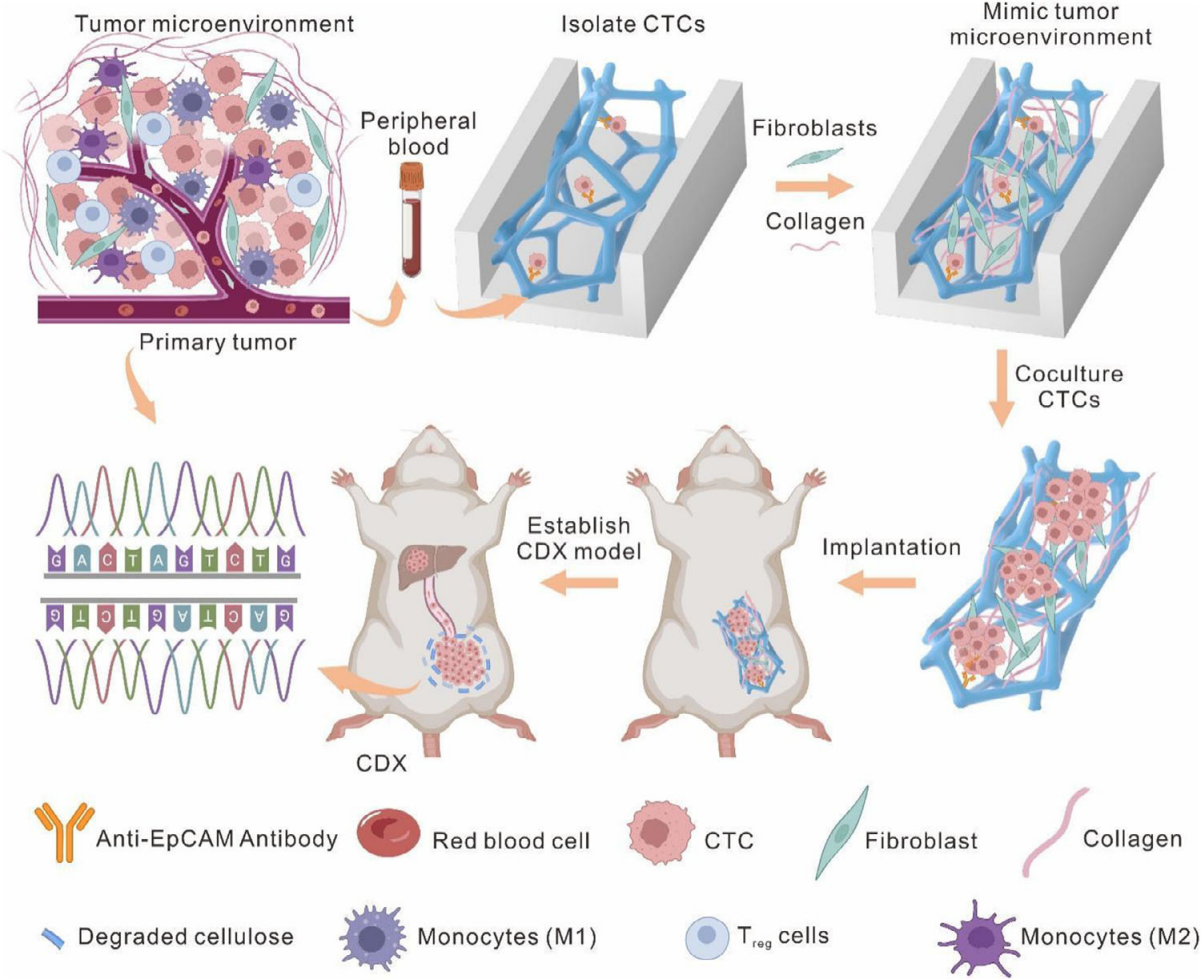
Schematic diagram showing the biodegradable 3D cellulose scaffold for capture, expansion, and in vivo application of circulating tumor cells (CTCs). First, the antibody‐functionalized cellulose scaffold was integrated into the customized microchip and used to capture CTCs from patients’ blood. Then, CTCs were co‐cultured with collagen and fibroblasts in the cellulose chip. Next, the cellulose scaffold with cultured CTCs was implanted into the immunodeficient mouse to construct the patient CTC‐derived xenograft (CDX) model for monitoring tumor metastasis. During this process, the cellulose scaffold gradually degraded. This diagram was created using BioRender.com.

To address these limitations, we have developed a novel 3D macroporous cellulose scaffold, enabling the capture and in situ culture of CTCs, followed by direct implantation into immunodeficient mice for CDX construction (Figure 1). The scaffold could be easily biofunctionalized and integrated into a microfluidic chip, and its interconnected macropores promoted chaotic flow for efficient CTC capture, while its flexibility minimized mechanical damage, preserving cell viability. Additionally, the scaffold was biocompatible and permeable to macromolecules, creating an optimal environment for the rapid expansion of CTCs, supported by collagen and stromal fibroblasts. Importantly, the scaffold could be easily retrieved from the microfluidic chip and implanted directly into mice, with the scaffold gradually degrading into glucose. This 3D macroporous cellulose scaffold has been successfully applied to capture and culture CTCs from breast cancer patients' blood, enabling the construction of CDX models. These models have proven effective in studying tumor metastasis and offer significant potential for personalized cancer therapy and oncology research.

## 2 Results

### 2.1 Preparation and Characterization of 3D Cellulose Scaffold

**FIGURE 2:**
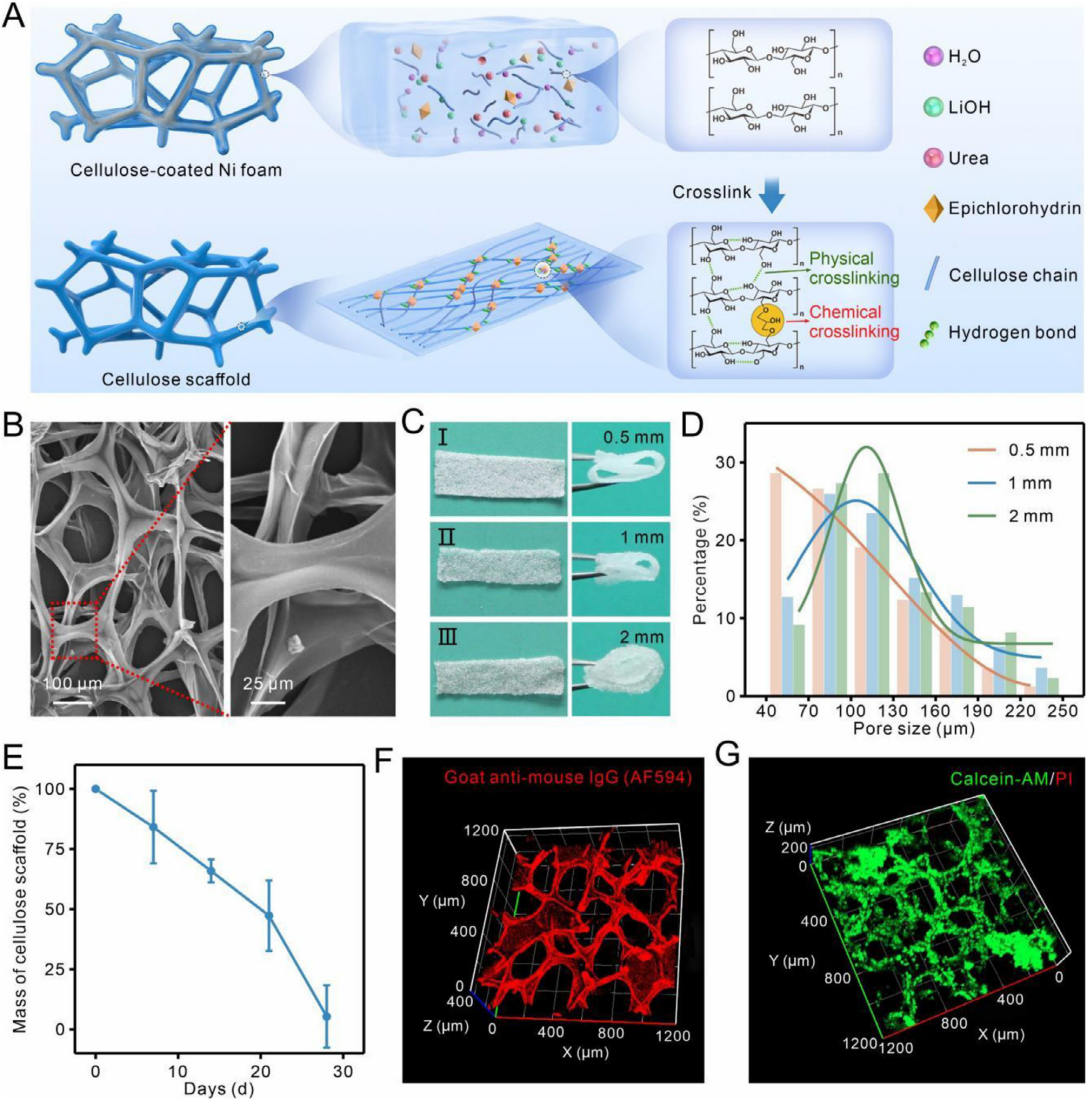
Characterization of 3D cellulose scaffold. (A) Diagram representing the cross‐linking mechanism of cellulose in the fabrication of 3D cellulose scaffold. (B) Scanning electron microscopy (SEM) images captured under different magnifications revealed the morphological structure of 3D cellulose scaffold fabricated by assembling four layers of cellulose on 1 mm‐thick Ni foam. (C) Photos of 3D cellulose scaffold fabricated from different thickness Ni foams under unfolding and folding. (I) Ni foam with 0.5 mm thickness; II) Ni foam with 1 mm thickness; III) Ni foam with 2 mm thickness. (D) Column chart representing pore size distribution of varied 3D cellulose scaffolds fabricated from Ni foams with different thicknesses (orange, 0.5 mm, n = 252 pores; blue, 1 mm, n = 363 pores; green, 2 mm, n = 308 pores). The solid line showing the result obtained by fitting the Gauss function. (E) Degradation curve of cellulose scaffold immersed in cellulase solution during one month (n = 3, data were shown as the mean ± standard deviation (SD)). (F) Laser scanning confocal microscopy (LSCM) image of anti‐epithelial cell adhesion molecule (anti‐EpCAM) antibody‐functionalized cellulose scaffold characterized by DyLight 594‐labeled goat anti‐mouse secondary antibody. (G) LSCM image of MCF‐7 cells cultured in 3D cellulose scaffold for 7 days and stained by Calcein‐AM (green) and propidium iodide (PI, red).

The cotton linter pulp, a rich source of cellulose, was dissolved in a lithium hydroxide (LiOH)/urea aqueous solution at low temperature to disrupt the hydrogen bonds between cellulose molecular chains and form a uniform viscous cellulose solution [30, 31, 32, 33]. As‐prepared cellulose solution was then spin‐coated onto a nickel (Ni) foam skeleton via centrifugation, followed by solidification in water through the reformation of hydrogen bonds by physical cross‐linking (Figure S1A). To further enhance the structural integrity, epichlorohydrin (ECH) was added to the cellulose solution to chemically cross‐link the hydroxyl groups between cellulose chains. This combination of hydrogen and chemical bonds improved the interaction between cellulose chains, enhancing the mechanical properties of the scaffold (Figure 2A).

To produce a cellulose scaffold with intact, continuous, and self‐supporting macroporous structure, multiple layers of cellulose were spin‐coated onto the Ni foam. Subsequently, the Ni foam was etched away using nitric acid (HNO_3_), leaving behind a cross‐linked cellulose scaffold (Figure S1B–D; Figure 2B). As shown in Figure 2C and Figure S2, various cellulose scaffolds that varied in thickness and macropore size distribution were fabricated with excellent flexibility and integrity even under extreme bending and folding conditions. The Young's modulus and fracture energy of the cellulose scaffold was calculated as 294.4 kPa and 201.6 kJ m^−3^, based on the stress‐strain curve (Figure S3), demonstrating suitable mechanical properties for further CTC capture and in vivo implantation. Quantitative analysis of scanning electron microscopy (SEM) images revealed that macropores ranged in size from 40 to 250 µm, with thinner scaffolds containing smaller pores and thicker ones having a larger proportion of bigger pores (Figure 2D). Thus, a flexible, self‐supporting cellulose scaffold with adjustable macropores was successfully developed.

Although cross‐linked, the cellulose scaffold retained its biodegradability when immersed in a cellulase solution. As shown in Figure 2E and Figure S4, the scaffold's size and weight gradually decreased, disappearing entirely within 30 days, confirming that the 3D cellulose scaffold maintained the biological properties and biodegradability of cellulose. To modify the scaffold, anti‐epithelial cell adhesion molecule (anti‐EpCAM) antibodies were conjugated onto the surface, and the presence of the antibodies was confirmed using DyLight 594‐labeled goat anti‐mouse secondary antibodies. Fluorescence imaging (Figure 2F) showed strong red fluorescence, outlining the interconnected macroporous structure of the scaffold. In contrast, scaffolds without anti‐EpCAM antibodies exhibited minimal red fluorescence (Figure S5), confirming the successful and uniform conjugation of anti‐EpCAM antibodies for specific recognition of CTCs.

To evaluate biocompatibility, cancer cells and fibroblasts were cultured on the cellulose scaffold for seven days and stained with Calcein‐AM and propidium iodide (PI). As shown in Figure 2G and Figure S6, almost all cells exhibited strong green fluorescence, indicating good bioactivity and adherence to the scaffold, even under harsh conditions such as phosphate‐buffered saline (PBS) flushing. These results demonstrated the excellent biocompatibility and proliferation support of the cellulose scaffold.

### 2.2 Performance of 3D Cellulose Scaffold Microchip for Capturing CTCs

**FIGURE 3:**
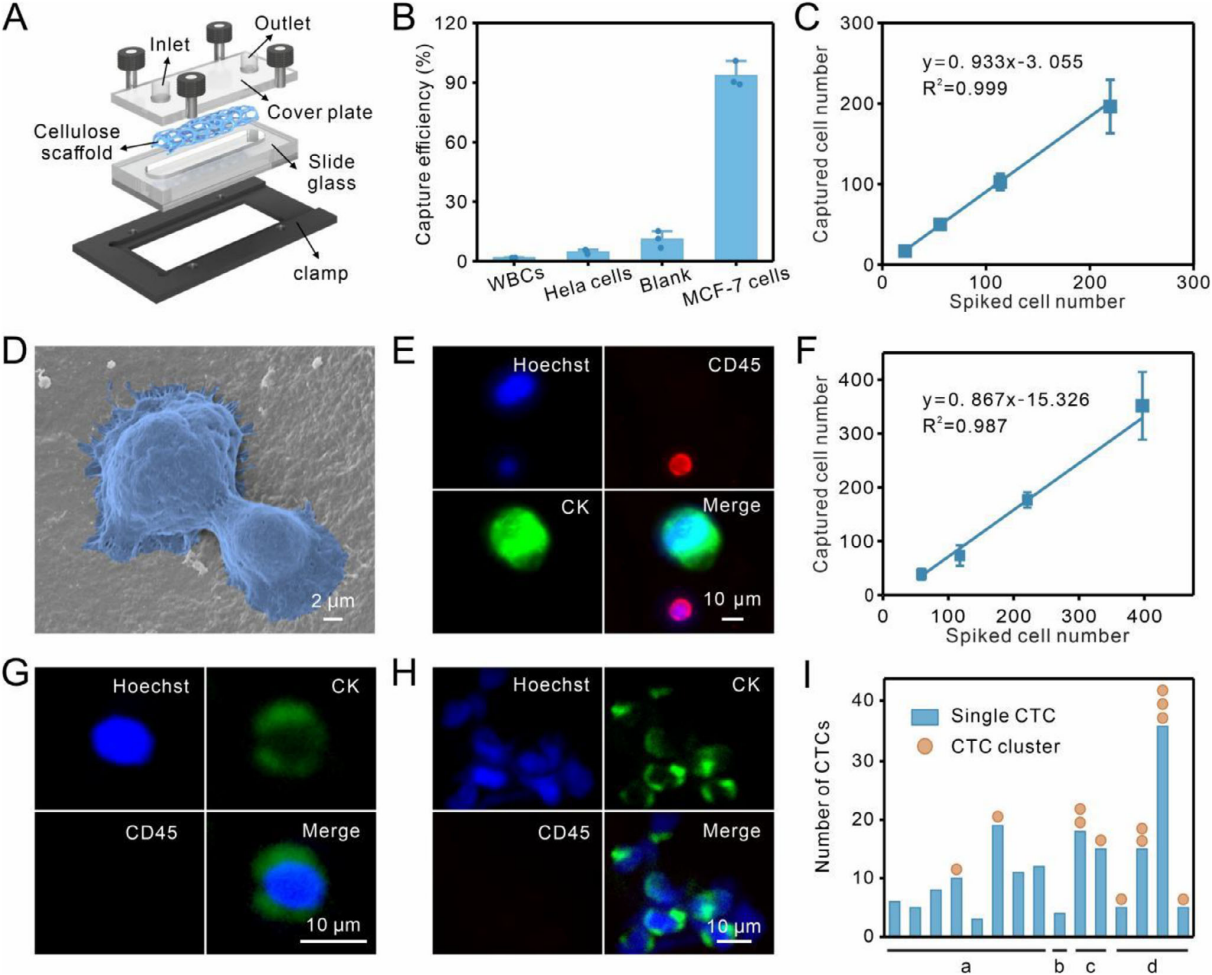
Capture of CTCs by 3D cellulose scaffold microchip. (A) Schematic diagram illustrating the components of the 3D cellulose scaffold microchip. (B) Comparison of capture efficiency among WBCs, HeLa cells, and MCF‐7 cells using the cellulose chip. The blank data point represented MCF‐7 cells captured in the cellulose chip without immobilization of anti‐EpCAM antibodies (n = 3, mean ± SD). (C) Quantitative capture efficiency of MCF‐7 cells spiked in PBS (n = 3, mean ± SD). (D) SEM image of tumor cells captured by the cellulose chip. (E) Representative fluorescence images of WBCs (Hoechst+/CK‐/CD45+) and cancer cells (Hoechst+/CK+/CD45‐) captured by the cellulose chip from mimic cancer patient's blood. (F) Capture efficiency of MCF‐7 cells spiked in human blood (n = 3, mean ± SD). (G,H) Representative fluorescence images showing a single CTC (G) and a CTC cluster (H, Hoechst+/CD45‐/CK+) captured by the cellulose chip from the blood of cancer patients. (I) Quantitative data depicting the number of CTCs and CTC clusters captured from the blood of cancer patients (a: lung cancer; b: nasopharyngeal cancer; c: esophageal cancer; d: gastric cancer).

The anti‐EpCAM antibody‐functionalized cellulose scaffold was integrated into a customized single‐channel manifold and assembled into a microfluidic chip (Figure 3A; Figures S7 and S8). To assess the chip's performance, MCF‐7 cells, which overexpressed EpCAM, were selected as model cancer cells, while HeLa cells and white blood cells (WBCs) were used as negative controls. Quantitative analysis revealed that only 11.1% of MCF‐7 cells were non‐specifically trapped in the cellulose chip without anti‐EpCAM antibody. However, when the chip was conjugated with the anti‐EpCAM antibody, the capture efficiency significantly increased to 93.3%. In contrast, the capture efficiencies for HeLa cells and WBCs were only 4.8% and 1.8%, respectively (Figure 3B; Figure S9).

Figure 3C illustrated that the chip could efficiently capture MCF‐7 cells in a range from 20 to 400 cells spiked in PBS. SEM imaging further revealed that the captured MCF‐7 cells extended filopodia and adhered to the cellulose scaffold, showing localized topographical interactions between the cells and the scaffold (Figure 3D). These results confirmed the specific and efficient capture of CTCs using the cellulose chip.

To simulate the capture of CTCs from cancer patient blood, MCF‐7 cells were spiked into healthy human blood and injected into the cellulose chip. As shown in Figure 3E, cancer cells were successfully captured and identified using three‐color immunocytochemistry (ICC), targeting cytokeratin (CK, green), CD45 (red), and the nucleus (Hoechst 33258, blue). MCF‐7 cells were characterized by Hoechst+/CK+/CD45‐ staining and were larger than 10 µm, whereas WBCs were identified as Hoechst+/CK‐/CD45+ and were smaller than 10 µm. Quantitative analysis demonstrated that the cellulose chip achieved a capture efficiency of 86.7% for MCF‐7 cells, even at varying concentrations of 50 to 400 cells per 1 mL of blood (Figure 3F). Next, as shown in Figure 3G–I and Table S1, immunostaining and quantitative data showed that both individual CTCs and CTC clusters were efficiently captured from blood samples of real clinical cancer patients, laying the groundwork for subsequent in vitro culture. These results highlighted the chip's potential for efficiently isolating CTCs from cancer patient blood samples, making it suitable for clinical applications in cancer research and diagnostics.

### 2.3 Proliferation of Captured CTCs in the 3D Cellulose Scaffold Microchip

**FIGURE 4:**
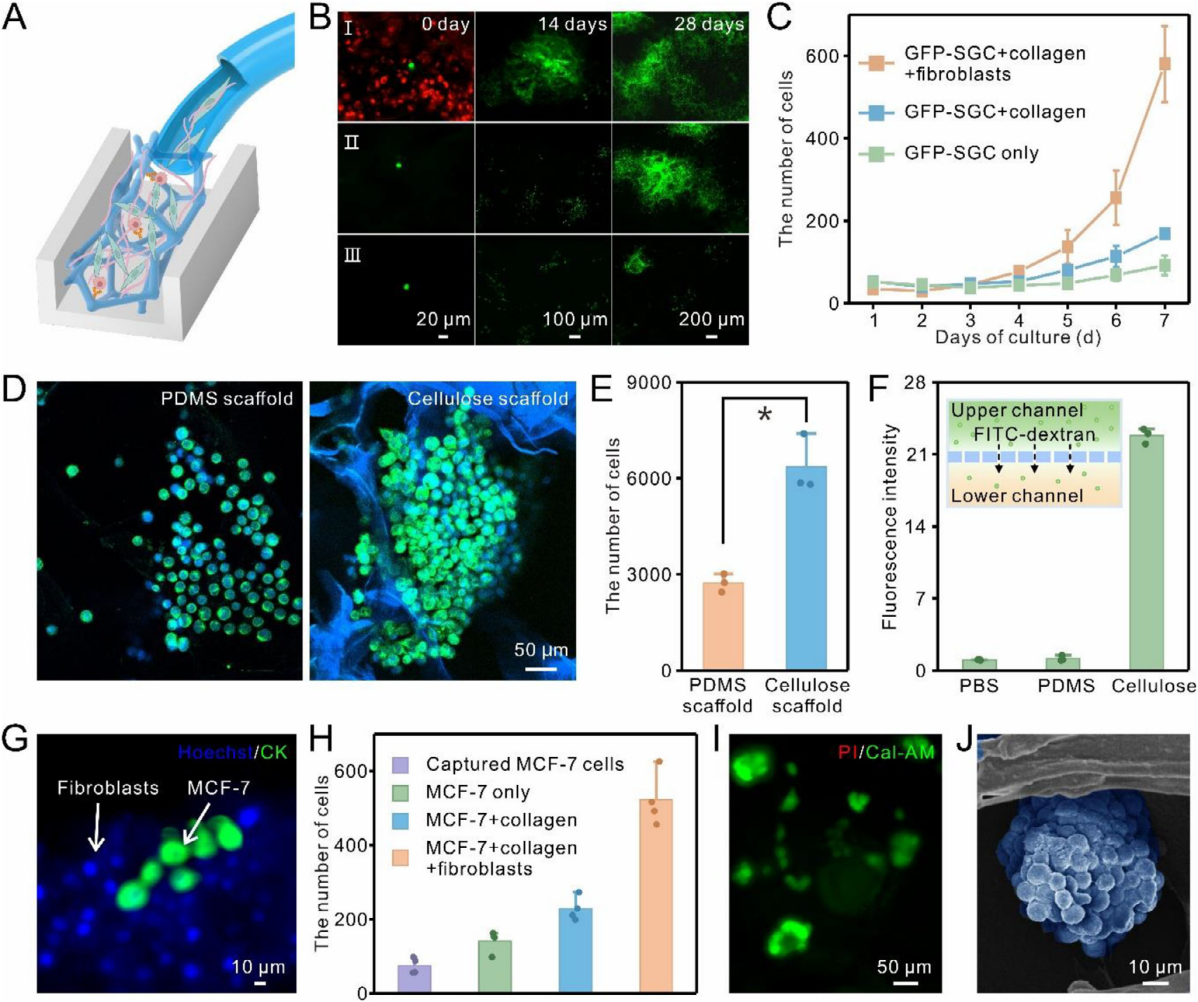
Expansion of CTCs in the cellulose scaffold microchip. (A) Schematic diagram illustrating the introduction of fibroblasts and collagen into the microchip to facilitate the expansion of captured CTCs. This diagram was created using BioRender.com. (B) Fluorescence images of GFP‐SGC cells (green) cultured in the chip under three conditions for 28 days: (I) co‐cultured with collagen and DiI‐labeled fibroblasts (red), (II) cultured only with collagen, (III) cultured individually. (C) Proliferation curves of GFP‐SGC cells cultured in the chip for the first 7 days (n = 3, mean ± SD). (D,E) Representative fluorescence images (D) and quantitative data (E) of MCF‐7 cells (Hoechst+/CK+) after a 14‐day co‐culture in a polydimethylsiloxane (PDMS) scaffold chip versus a cellulose scaffold chip (n = 3, mean ± SD; two‐tailed t‐test, *p < 0.05). (F) Normalized fluorescence intensity of the solution from the lower channel in the dual‐channel microfluidic device, integrating either PDMS or cellulose as a mid‐layer (n = 3, mean ± SD). The inset depicted the diffusion of FITC‐dextran through the mid‐layer cellulose membrane in the device. (G,H) Representative fluorescence image (G) and quantitative data (H) of MCF‐7 cells after capture from mimic cancer patient blood and co‐culture for 7 days in the chip (n = 4, mean ± SD). (I) Fluorescence image demonstrating the bioactivity of MCF‐7 cells after capture and co‐culture in the chip for 7 days, with cultured cells stained using Calcein‐AM (green) and PI (red). (J) SEM image of MCF‐7 cell clusters acquired through co‐culture in the chip.

The extracellular matrix and stromal cells within the tumor microenvironment play a crucial role in cancer cell proliferation [34, 35, 36]. Therefore, collagen and fibroblasts were incorporated into the cellulose scaffold chip to enable successful in situ expansion of captured CTCs (Figure 4A). Here, EpCAM‐positive and green fluorescent protein (GFP)‐transfected human gastric adenocarcinoma (GFP‐SGC) cells were selected as model CTCs and cultured in the cellulose chip under varying conditions for cell proliferation visualization. As shown in Figure 4B, GFP‐SGC cells cultured solely in the presence of collagen had an improved proliferation rate compared with those cultured without collagen. In contrast, when co‐cultured with collagen and fibroblasts, GFP‐SGC cells rapidly proliferated by day 7 (Figure 4C) and continued to fill the entire 3D scaffold with prolonged cultivation over 28 days. Notably, the highest fold expansion of the captured cancer cells was achieved under co‐culture conditions with about 100 000 fibroblasts in the cellulose chip (Figure S10). This might be attributed to insufficient growth factor secretion at low fibroblast density and potential nutrient competition with cancer cells at excessively high fibroblast density. These results suggested that fibroblasts, through the secretion of growth factors and collagen, as part of the extracellular matrix, synergistically promoted the proliferation of cancer cells in the cellulose microchip.

The compatibility of cellulose scaffold for CTC culture was further proved by monitoring the proliferation of MCF‐7 cells in cellulose, which was compared concurrently with polydimethylsiloxane (PDMS) scaffold‐based cell culture. Figure 4D,E demonstrated that cells cultured in the cellulose scaffold exhibited faster growth and more compact clustering than those in the PDMS scaffold, which might be due to the different permeability of these two scaffolds. Therefore, to prove our supposition, a flat cellulose membrane was prepared and integrated into a dual‐channel microfluidic device (Figure S11), with a PDMS membrane serving as the control. Macromolecular FITC‐dextran and PBS were introduced into the upper and lower channels, respectively. After 1 h, the fluorescence intensity in the lower channel of the cellulose membrane‐integrated device significantly increased (Figure 4F), indicating the diffusion of FITC‐dextran through the cellulose membrane—a phenomenon not observed in the PDMS membrane. Additionally, SEM imaging revealed the hollow structure of the cellulose scaffold, resulting from the complete etching of the Ni foam template (Figure S12). These findings underscored the scaffold's excellent macromolecular permeability and hollow structure, which facilitated nutrient transport in confined spaces and thereby enhanced tumor cell growth.

A mimic blood sample (1 mL of blood spiked with 100 MCF‐7 cells) was further prepared to evaluate the performance of cellulose scaffold for capture and in situ culture of CTCs from blood. Immunostaining and quantitative analysis showed that MCF‐7 cells (Hoechst+/CK+) proliferated 7.0‐fold when co‐cultured with collagen and fibroblasts (Hoechst+/CK‐), compared to 3.0‐fold with collagen alone and 1.9‐fold without collagen (Figure 4G,H). These results confirmed that fibroblasts and collagen synergistically promoted the proliferation of CTCs. Moreover, MCF‐7 cells displayed high bioactivity after 7‐day proliferation (Figure 4I). SEM images also showed that MCF‐7 cells formed tumor spheres suspended on the cellulose scaffold (Figure 4J). Collectively, these findings highlighted that the 3D cellulose microchip not only efficiently isolated CTCs from whole blood but also supported their proliferation. Notably, the CTCs adhered to the cellulose scaffold and formed tumor spheres, maintaining their anchorage‐independent phenotype, a key characteristic of CTCs.

### 2.4 Validation of the 3D Cellulose Scaffold for Tumor Xenograft Construction

**FIGURE 5:**
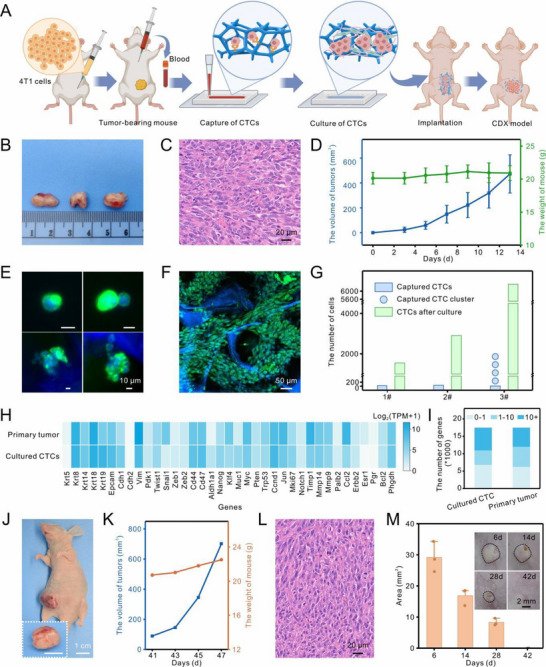
Capture, culture, and implantation of CTCs from whole blood of tumor‐bearing mice. (A) Schematic diagram illustrating the experimental procedure for capturing, culturing, and implanting CTCs from tumor‐bearing mice. This diagram was created using BioRender.com. (B) Representative images of tumors obtained from tumor‐bearing Balb/c mice. (C) Hematoxylin and eosin (H&E) staining of tumor tissue from the tumor‐bearing mouse. (D) Curves depicting tumor growth and weight change in mice following injection with 4T1 cells (n = 18, mean ± SD). (E) Representative fluorescence images of single CTCs and CTC clusters captured from the blood of tumor‐bearing mice. (F) Fluorescence images of CTCs cultured in a 3D cellulose scaffold chip for two months. CTCs were identified as Hoechst+/CK+/CD45‐ and larger than 10 µm. (G) Quantitative data showing the number of CTCs captured and co‐cultured using the cellulose chip from whole blood of three tumor‐bearing mice. (H) Heat map displaying expression levels of key genes in cultured CTCs compared to primary tumor cells. (I) Statistical map indicating the number of genes at different expression levels in the two samples. (J) Images of tumor growth in a Balb/c nude mouse after implantation with the cellulose scaffold containing cultured CTCs. (K) Curves illustrating tumor growth and weight change in this nude mouse post‐implantation. (L) H&E staining of tumor tissue from the nude mouse. (M) Changes in the hump area over time at the implantation site of the sterile, cell‐free cellulose scaffold in Balb/c nude mice, with insets showing images of the implantation site. The black dotted line indicated the hump area after cellulose scaffold implantation (n = 3, mean ± SD).

Tumor‐bearing mice were constructed to provide the fresh whole blood for processing by 3D cellulose scaffold for CTC capture, in situ culture, and direct implantation to establish tumor xenografts, which was illustrated in Figure 5A. Tumor growth and histopathological analysis confirmed the successful establishment of tumor‐bearing mice (Figure 5B–D; Figure S13). Subsequently, the blood was extracted from the mice and injected into the 3D cellulose scaffold embedded microchip. The CTCs, including both individual cells and clusters, were effectively captured by the scaffold and identified through immunostaining for CK (green), CD45 (red), and nuclear Hoechst staining (blue) (Figure 5E). Upon co‐culture with collagen and fibroblasts in the microchip, the captured CTCs proliferated significantly, forming larger cell aggregates (Figure 5F). Quantitative analysis demonstrated that the CTC population expanded by approximately 45.5‐ to 81.3‐fold during the culture period (Figure 5G). These findings highlighted the scaffold's efficiency in isolating CTCs from whole blood and its capacity to support their in situ expansion.

To further characterize the expanded CTCs, single‐cell transcriptome sequencing was performed. Gene expression profiling revealed a high degree of similarity between the cultured CTCs and primary tumor cells derived from the tumor‐bearing mice, with a Pearson correlation coefficient of approximately 0.85 (Figure S14). Key epithelial (keratin 5 (Krt5), keratin 8 (Krt8), keratin 14 (Krt14), keratin 18 (Krt18), Epcam, Cadherin 1 (Cdh1)) and mesenchymal markers (Cadherin 2 (Cdh2), Vimentin (Vim), pyruvate dehydrogenase kinase 1 (Pdk1)), stem cell‐associated genes (Cd44, Cd47, aldehyde dehydrogenase 1 family member A1 (Aldh1a1), nanog homeobox (Nanog)), and genes involved in proliferation and cell cycle regulation (cyclin D1 (Ccnd1), jun proto‐oncogene (Jun), Ki67 (Mki67), notch receptor 1 (Notch1)) exhibited comparable expression levels between the two cell types (Figure 5H,I).

Following the in vitro expansion, the cultured CTCs, together with the 3D cellulose scaffold, were implanted into the mammary gland of a separate nude mouse (Figure 5A). Over time, a distinct tumor mass became apparent at the implantation site, demonstrating rapid growth (Figure 5J,K). Histopathological analysis of the excised tumor tissue revealed an abundance of tumor cells, which were positively stained for the epithelial cancer marker cytokeratin (CK) and the proliferation marker Ki67 (Figure 5L; Figure S15). These findings confirmed the successful establishment of a robust CDX model.

In addition to supporting tumor growth, the cellulose scaffold exhibited gradual biodegradation throughout the experimental timeline. To prove this phenomenon, a cell‐free cellulose scaffold was implanted into the mammary gland of nude mice as a control (Figure S16). For more than one month, the scaffold‐based prominence progressively diminished, eventually becoming undetectable (Figure 5M). This observation aligns with the literature reporting that numerous potential cellulose‐degrading microorganisms exist in the animals and are likely responsible for the scaffold's disappearance [37, 38]. Histological analysis of tissues surrounding the implanted sterile scaffold showed no significant signs of tissue damage or adverse reactions, further underscoring the scaffold's biocompatibility (Figure S17).

These findings collectively validated the 3D cellulose scaffold as a biocompatible and biodegradable platform for in vivo applications. The scaffold integrated key processes—CTC capture, in situ culture, and subsequent implantation—into a streamlined approach, enabling the efficient establishment of tumor xenografts. This innovative method represented a significant advancement in cancer research, providing a versatile tool for studying tumor progression, metastasis, and personalized therapeutic responses in vivo.

### 2.5 Constructing Patient‐Derived Xenografts by CTCs From Cancer Patient Blood

**FIGURE 6:**
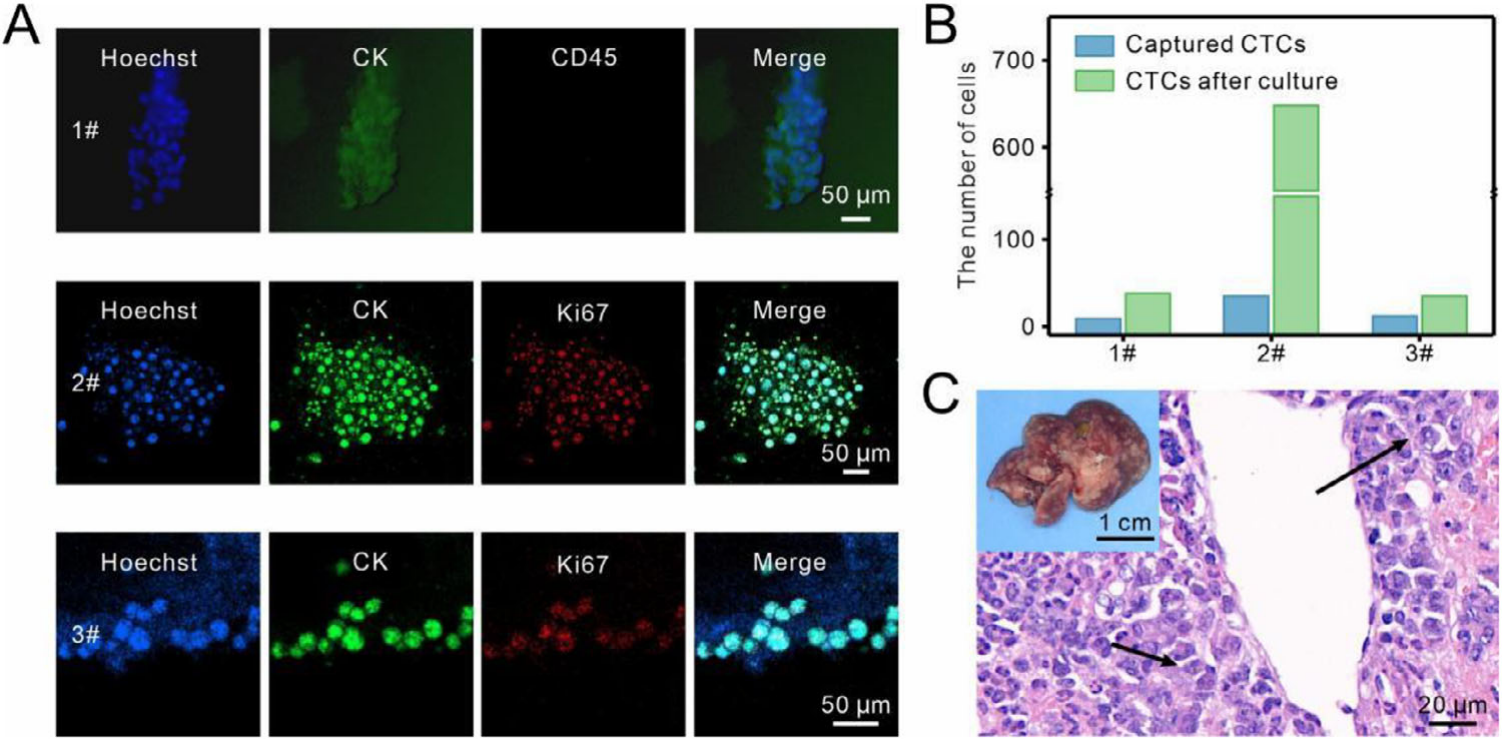
Capture, culture, and in vivo implantation of CTCs from cancer patient blood. (A) Representative fluorescence images of CTCs from three breast cancer patients (1#, 2#, and 3#) after capture and co‐culture in the cellulose chip. (B) Quantitative data of CTCs following capture and co‐culture in the cellulose chip. (C) H&E staining of liver tissue from a Balb/c nude mouse implanted with the cellulose scaffold containing cultured CTCs from breast cancer patient 2#. Black arrows indicated cancer cells in the liver, with the inset showing a detailed view of the liver tissue.

The 3D cellulose scaffold microchip was ultimately applied to clinical cancer patients' blood samples. Fresh blood samples from three breast cancer patients were processed through the cellulose scaffold microchip and co‐cultured with fibroblasts and collagen. As illustrated in Figure 6A, Figures S18 and S19, the captured CTCs and CTC clusters expanded into larger clusters over time within the scaffold. The expanded cells expressed high levels of cancer‐specific marker CK and proliferation marker Ki67, while remaining negative for the leukocyte marker CD45. Quantitative analysis revealed that the number of cancer cells increased 2.9‐ to 18.5‐fold following culture within the chip (Figure 6B; Table S2).

Subsequently, the cellulose scaffold containing expanded CTCs from a breast cancer patient was implanted into the mammary gland of a mouse. Over time, the mice exhibited a slight increase in body weight without adverse behavioral effects (Figure S20). Histological examination via H&E staining (Figure 6C) and immunostaining (Figure S21) using a human‐specific Ki67 antibody confirmed the presence of proliferative, human‐derived metastatic cancer cells in the liver of xenografted mice. The CTCs used in this implantation originated from patient 2#, who had been diagnosed with invasive breast cancer with a high histologic grade. Consequently, the high proliferation rate of CTCs in the scaffold and their aggressive metastatic behavior were observed. In contrast, tumors from the other two patients (patients 1# and 3#), with less invasive disease, exhibited slower growth rates and fewer metastases.

These findings suggested that the 3D cellulose scaffold enabled efficient isolation and expansion of CTCs from patient blood and supported direct implantation for constructing xenograft models that replicated the invasive characteristics of a patient's tumor. Compared with other CTC culture methods, this cellulose scaffold‐based system offered a high success rate for CTC proliferation and maintained excellent bioactivity for promoting in vivo metastasis. The self‐supporting, biocompatible, and biodegradable properties of the cellulose scaffold facilitated the integration of CTC capture, culture, and direct implantation, providing a powerful platform for cancer research and personalized anticancer therapy.

## 3 Conclusion

PDX models have emerged as indispensable tools in tumor biology research and the advancement of personalized cancer therapies [1, 2]. CTCs, which serve as critical biomarkers in liquid biopsy, provide a minimally invasive avenue for the development of CDX models. These models bridge the gap between patient‐specific tumor biology and clinical applications, offering an essential platform for translational cancer research [9, 12, 14]. The rarity and low viability of CTCs necessitate highly efficient and mild capture techniques, while traditional isolation methods often introduce physical stress that compromises cell viability and integrity [15, 16, 17]. The loss of the tumor microenvironment further limits CTC survival and proliferation in vitro, hindering their tumorigenic potential [18, 19, 20]. Furthermore, due to the limitations of cell culture by microchips, it is unsuitable to direct implantation of the obtained CTCs for CDX establishment [26, 27, 28, 29].

To address these limitations, we developed an innovative strategy centered on a 3D macroporous cellulose scaffold that facilitates efficient CTC capture, in situ culture, and subsequent CDX model construction. Unlike conventional CTC capture methods that involve labor‐intensive separation steps and compromise cell viability [10, 18, 19, 20], our scaffold retained flexibility and a highly interconnected macroporous architecture, which minimized mechanical stress and provided robust support. When integrated into a microfluidic chip, the scaffold enabled efficient CTC isolation directly from whole blood, achieving a capture efficiency of 86.7% while preserving cellular integrity.

Tumor progression and metastasis are profoundly influenced by interactions between tumor cells and the surrounding microenvironment, including stromal fibroblasts, extracellular matrix components, and immune cells. Recognizing this, we incorporated fibroblasts and collagen into the cellulose scaffold to simulate the tumor microenvironment, thereby enhancing the proliferation and viability of isolated CTCs. Our results demonstrated that co‐culture with these microenvironmental components significantly promoted CTC expansion, enabling their transformation into tumorigenic cell clusters. Moreover, the biocompatibility and permeability of cellulose further supported the rapid in situ proliferation of captured CTCs within the microchip. Traditional microfluidic‐based platforms, while effective for in vitro tumor studies, often rely on materials that lack in vivo degradability and exhibit biological toxicity, making them unsuitable for direct implantation for constructing a CDX model [26, 28, 29]. Our findings highlighted that the scaffold with excellent biodegradability ensured compatibility with in vivo applications and minimized the risk of long‐term adverse effects, facilitating seamless implantation of obtained CTCs and the efficient construction of CDX models. When scaffolds containing patient‐derived CTC spheres were implanted into mice, clear tumor metastasis was observed, validating the feasibility of this approach for studying tumor biology in vivo.

This study presents a straightforward yet powerful method for capturing, culturing, and applying patient‐derived CTCs, marking a significant advancement in CTC‐based cancer research. By leveraging the 3D macroporous cellulose scaffold, we have substantially improved CTC isolation efficiency and maintains CTC viability. The inclusion of co‐culture elements effectively simulates the tumor microenvironment, promoting the expansion of CTCs. Furthermore, the biocompatible and degradable scaffold provides an ideal material for in vivo implantation of cultured CTCs.

Despite the robust performance of 3D cellulose scaffold demonstrated in this work, the EpCAM‐based CTC capture has its limitations since the expression of EpCAM can be downregulated in certain CTC subpopulations, particularly those undergoing epithelial‐mesenchymal transition (EMT), which may result in incomplete recovery of the full CTC spectrum. To address this issue, the versatile surface chemistry of the cellulose scaffold provides a flexible platform for further functionalization. In future developments, the scaffold could be modified with a cocktail of antibodies recognizing epithelial (EpCAM, epidermal growth factor receptor), mesenchymal (vimentin, N‐cadherin), and cancer stem cell markers (CD44, CD133) to enable simultaneous capture of heterogeneous CTC phenotypes.

It is noted that the macroporous and flexible nature of the cellulose scaffold provides a physical obstructing effect favorable for label‐free isolation of CTC clusters [39]. And the 3D cellulose scaffold microchip could also be easily integrated with label‐free enrichment methods that exploit the intrinsic biophysical differences between CTCs and blood cells, such as size, deformability, and dielectric properties, to enrich and culture marker‐independent CTCs. Such a multi‐modal approach would broaden the clinical applicability of the platform and facilitate the creation of physiologically relevant tumor models for precision oncology.

## 4 Experimental Section

### 4.1 Materials and Instruments

Nickel (Ni) foams (0.5–2 mm in thickness) were purchased from Suzhou Taili Electronics Co., Ltd. Cellulose sample (cotton linter pulp) was supplied by Hubei Chemical Fiber Group Ltd. from Xiangyang. Lithium hydroxide monohydrate (LiOH·H_2_O), urea, epichlorohydrin (ECH), nitric acid (HNO_3_), sodium hydroxide (NaOH) and ethanol were obtained from Sinopharm Chemical Reagent Co., Ltd. Streptavidin (SA), N‐(3‐dimethylaminopropyl)‐N’‐ethylcarbodiimide hydrochloride (EDC), N‐Hydroxysuccinimide (NHS), Calcein‐AM, propidium iodide (PI), bovine serum albumin (BSA), FITC‐dextran (40 KDa) and Hoechst 33258 were bought from Sigma‐Aldrich. Carboxyethylsilanetriol disodium salt was supplied by Gelest Inc. Cellulase solution was obtained from Aladdin Industrial Co., Ltd. Collagenase I and 4% paraformaldehyde were obtained from Biosharp. 1,1‐Dioctadecyl‐3,3,3,3‐tetramethylindocarbocyanine perchlorate (DiI) and biotin‐labeled anti‐EpCAM monoclonal antibody were obtained from ThermoFisher. Triton X‐100 was purchased from Beyotime Biotechnology. DyLight 594‐labeled goat anti‐mouse secondary antibody, Alexa Fluor 488‐labeled anti‐pan cytokeratin antibody (AF488‐CK), PE‐labeled anti‐CD45 antibody (PE‐CD45), and Alexa Fluor 647‐labeled anti‐Ki67 antibody (AF647‐Ki67) were purchased from Abcam Company. MCF‐7 cells (human breast cancer cells), NIH/3T3 cells (mouse embryonic fibroblast cells), 4T1 cells (mouse breast cancer cells), and Hela cells (human cervical cancer cells) were bought from China Type Culture Collection. Human mammary fibroblast cell line (HMF7630 cells) was purchased from Shanghai Qingqi Biotechnology Development Co., Ltd. GFP‐SGC7901 cells (human gastric adenocarcinoma cells) were obtained from Renmin Hospital of Wuhan University. All the related reagents used for cell culture were purchased from Gibco Corp. Collagen solution (Collagen type I, rat tail, 4.16 mg mL^−1^) was obtained from Corning. The customized microchip with single channel manifold (the geometric size: 25 mm × 4 mm × 0.6 mm, length × width × thickness) was supplied by Wuhan MesoBiosystem Co., Ltd. Blood samples were obtained from Renmin Hospital and Zhongnan Hospital of Wuhan University.

SEM images were taken by using a field‐emission scanning electron microscope (TESCAN CLARA, Czech Republic). Fluorescent microscopic images were obtained using a Zeiss microscope (AxioObserver Z1, Zeiss, Germany). Laser scanning confocal microscopy images were taken by ZEISS LSM 900.

### 4.2 Preparation of 3D Cellulose Scaffold

First of all, the cellulose solution was prepared in according to the method detailed in the previous work [30, 31, 40, 41]. In brief, 8 g LiOH·H_2_O and 15 g urea were added to 77 g ultrapure water, which were ultrasonically dispersed to obtain a transparent LiOH/urea aqueous solution. Then, 4 g cotton linter pulp full of cellulose was completely immersed in the as‐prepared LiOH/urea solution, and then frozen at −40 C for 5 h. When thawing at room temperature, a clear 4% cellulose solution was obtained.

The ECH and cellulose solution were mixed at a mass ratio of 1:27, stirred thoroughly, and poured into a 2 mL centrifuge tube. Subsequently, the cleaned Ni foam was placed in this centrifuge tube and centrifuged at 9000 rpm for 4 min. The Ni foam stuffed with the mixture of cellulose and ECH was then placed in another empty centrifuge tube for 3‐min centrifugation at 3000 rpm. Following, the cellulose‐coated Ni foam was immersed in ultrapure water for 30 min and then heated at 70 C for 2 h to make cellulose cross‐link. The above steps were repeated four times. The obtained cellulose‐coated Ni foam was subsequently soaked in 7 mol L^−1^ HNO_3_ for 3 h to etch Ni foam template. Next, it was placed in ultrapure water for 24 h to completely remove extra HNO_3_ and absorbed metal ions. Thus, the 3D cellulose scaffold was acquired. Ni foams of different thicknesses were used as templates to prepare 3D cellulose scaffolds, characterizing the structure of these scaffolds by SEM. More than 300 pores were calculated in each type of cellulose scaffold by Nano Measurer, and their pore size distributions were obtained. Then, the pore size distribution data were fitted with a Gaussian function using Origin 2019b. The fitting was performed via the nonlinear least‐squares method (Levenberg‐Marquardt algorithm).

To further characterize the mechanical properties of the cellulose scaffold, uniaxial tensile testing was applied. The 3D cellulose scaffold (2 cm in length, 0.8 cm in width) was affixed to the tensile stage and stretched at a rate of 5 mm min^−1^ until fracture was visually observed. The tensile distance was recorded to plot the stress‐strain curve. Then, the fracture energy was obtained by integrating the area under the curve, and Young's modulus was calculated according to the following equation:

$$E=\sigma /\varepsilon =\left(F/A\right)/\left(\Delta L/L_{0}\right)=FL_{0}/A\Delta L$$

where *E* is the Young's modulus, *σ* is the stress, *ε* is the strain, *F* is the tension, *A* is the area perpendicular to the tension, *ΔL* is the relative elongation length of the cellulose scaffold, and *L_0_* is the original length of the cellulose scaffold.

### 4.3 Characterization of 3D Cellulose Scaffold

First, the 3D cellulose scaffold was treated with plasma cleaner for 5 min, and then immersed in 5% carboxyethylsilanetriol disodium salt solution for 4 h to couple the carboxyl groups. Following, the carboxyl‐functionalized scaffold was washed with PBS three times. Second, the scaffold was activated by the solution containing 10 mmol L^−1^ EDC and 20 mmol L^−1^ NHS and then washed with PBS. Thirdly, the scaffold was incubated with 50 µg mL^−1^ SA solution for 4 h and then washed with PBS. Finally, 10 µg mL^−1^ biotin‐labeled mouse anti‐human anti‐EpCAM monoclonal antibody was incubated with the scaffold for 1 h. After washing with PBS, the antibody‐functionalized scaffold can be stored at 4 C until being used.

To verify the successful modification of the antibody, the antibody‐functionalized scaffold was incubated with DyLight 594‐labeled goat anti‐mouse secondary antibody for 1 h. Subsequently, the scaffold was washed with PBS and observed by microscope. While the scaffold without modification of anti‐EpCAM antibody was used as a control.

To characterize the biocompatibility of the cellulose scaffold, 10^5^ NIH/3T3 cells, SGC cells, MCF‐7 cells, and 4T1 cells were separately incubated with cellulose scaffolds for 30 min, and they were cultured in a 37 C incubator for 7 days. After that, the cells cultured on the cellulose scaffold were stained by Calcein‐AM and PI for 30 min in the incubator, washed with PBS three times, and then observed by fluorescence microscope.

To characterize the biodegradation of the cellulose scaffold in vitro, 3D cellulose scaffolds were dipped in cellulase solution for one month. The weight and morphology of the cellulose scaffold were recorded at 7‐day intervals. The experiment was repeated three times, and the mass change curve of the cellulose scaffold was obtained.

Moreover, a dual‐channel microfluidic device was utilized to examine the permeability of cellulose membrane [42, 43]. A mixture of ECH and cellulose (w/w = 1:27) was spin‐coated on a glass slide to acquire the complanate cellulose membrane, which was immersed in ultrapure water and then heated at 70 C to cross‐link. The dual‐channel microfluidic device was obtained by bonding a cellulose membrane to two PDMS layers containing straight recessed microchannels. The upper channel was introduced into macromolecular FITC‐dextran (1 mg mL^−1^) and the lower channel was introduced into PBS. Then, after the device was placed for 1 h, the solution in the lower channel was extracted and imaged by fluorescence microscope. The fluorescence intensity was then calculated by ImageJ software and normalized. Pure PBS and solution from the lower channel of PDMS membrane‐integrated microfluidic device were used as control. All these experiments were repeated three times.

### 4.4 Evaluating Capture Performance of 3D Cellulose Scaffold Microchip

An anti‐EpCAM antibody‐functionalized cellulose scaffold was integrated with the customized microfluidic chip with single channel manifold to get the 3D cellulose scaffold microchip [44]. MCF‐7 cells were selected as model cancer cells to evaluate the capture performance of the 3D cellulose scaffold microchip. Hela cells and WBCs were used as controls. WBCs were acquired from lysed healthy human blood. Hela cells, WBCs, and MCF‐7 cells were all stained with DiI and resuspended at 10^6^ cells per mL in PBS, respectively. 1 mL PBS containing 10^6^ Hela cells or WBCs was introduced into the cellulose chips at a flow rate of 50 µL min^−1^, and then the chips were washed by PBS at a flow rate of 50 µL min^−1^ for 3 min. These cells captured on chips were counted under the fluorescence microscope, and the capture efficiencies were calculated. Meanwhile, 1 mL PBS containing 200 DiI‐stained MCF‐7 cells was pumped into the cellulose chip without anti‐EpCAM antibody modification to calculate the capture efficiency. After that, DiI‐stained MCF‐7 cells were diluted to 100 cells per mL, respectively. Different numbers of MCF‐7 cells (20, 50, 100, 200, and 400 cells) were resuspended in PBS and pumped into the cellulose chips at a flow rate of 50 µL min^−1^, following by PBS washing. Finally, the captured cells were observed by fluorescence microscope and counted to calculate the capture efficiency.

Subsequently, 1 mL of healthy human blood was spiked with 50–400 MCF‐7 cells (50, 100, 200, 400 cells), which were used as mimic cancer patient blood samples. Then, the mimic samples were passed through the antibody‐functionalized cellulose scaffold microchips at a flow rate of 50 µL min^−1^, and chips were rinsed with PBS at a flow rate of 50 µL min^−1^ for 5 min to remove the hematologic cells. After that, 4% paraformaldehyde was added to the microchips for 30 min treatment. After PBS washing at a flow rate of 50 µL min^−1^ for 3 min, these microchips were treated with Triton X‐100 for 10 min and then washed with PBS. Next, 5% BSA was introduced into these microchips to treat for 1 h to reduce the nonspecific binding of fluorescent dyes, followed by PBS washing. Finally, a mixture of Hoechst 33258, AF488‐CK, and PE‐CD45 was injected into these microchips for 2‐h incubation at 4 C. After washing these chips with PBS at a flow rate of 50 µL min^−1^ for 10 min, these microchips were observed by fluorescence microscope and calculate capture efficiency. Captured cancer cells were classified as Hoechst+/AF488‐CK+/PE‐CD45‐ and were larger than 10 µm in size. While WBCs were defined as Hoechst+/AF488‐CK‐/PE‐CD45+ with a size smaller than 10 µm.

This study involving human participants was conducted in accordance with the ethical standards of the institutional and national research committee and with the Declaration of Helsinki. The study protocol was reviewed and approved by the Institutional Review Board (IRB) of Renmin Hospital of Wuhan University (Approval No. WDRY2024‐K031). Written informed consent was obtained from all participating blood donors prior to sample collection. Ethylenediamine tetraacetic acid anticoagulated blood samples from real clinical cancer patients (including lung cancer, nasopharyngeal cancer, esophageal cancer, and gastric cancer) were acquired. 1 mL of cancer patient blood was pumped into an antibody‐functionalized cellulose scaffold microchip at a flow rate of 50 µL min^−1^, and PBS was used to rinse the microchip to get rid of hematologic cells. Captured CTCs in the microchip were, in turn, fixed with 4% paraformaldehyde, permeabilized by Triton X‐100, treated with 5% BSA, and stained using Hoechst 33258, PE‐CD45, and AF488‐CK. Then, captured CTCs were imaged and counted by fluorescence microscope, which were defined as Hoechst+/AF488‐CK+/PE‐CD45‐. While WBCs were marked by Hoechst+/AF488‐CK‐/PE‐CD45+.

### 4.5 Culture of CTCs in 3D Cellulose Scaffold Microchip

Before the following experiments, cellulose scaffolds and customized microchips were first sterilized by autoclaving. The cellulose scaffolds were functionalized with anti‐EpCAM antibody under sterile conditions and integrated with the customized microchips to prepare the sterile antibody‐functionalized cellulose scaffold microchips.

GFP‐SGC cells were used as model cancer cells to visualize cell proliferation on the cellulose scaffold chip in the presence/absence of collagen and fibroblasts. Several GFP‐SG cells were passed through an antibody‐functionalized cellulose scaffold chip at a flow rate of 50 µL min^−1^, following by washing with culture medium at a flow rate of 50 µL min^−1^ to remove non‐specifically trapped cancer cells. After that, the collagen solution was diluted to 1 mg mL^−1^ and its pH was adjusted to 7.2–7.4 using 0.2 m NaOH solution. 10^5^ fibroblasts (using NIH/3T3 cells here) were dispersed in this collagen solution and then were immediately added to the microchip that had captured GFP‐SG cells. The microchip filled with only 1 mg mL^−1^ collagen solution and the microchip with GFP‐SGC cells individually cultured were taken as control groups. Following, these microchips were placed in a 37 C incubator for 1 h to make the collagen solidify, added with culture medium, and cultured in an incubator for 28 days. The culture medium was changed every 12 h. The entire process was carried out on a sterile bench. During the first week, these microchips were observed by a fluorescence microscope to count the number of GFP‐SGC cells every 24 h. Afterward, fluorescence images of GFP‐SGC cells were taken every 7 days to record the cell proliferation. Then, according to the above steps, three collagen solutions (each at 1 mg mL^−1^) containing different numbers of NIH/3T3 cells (100, 1000, or 2000 cells per µL) were prepared and introduced into the cellulose microchip (100 µL per chip). These cellulose chips were pre‐loaded with an identical number of 4T1 cells, enabling their pre‐capture within these chips. After a two‐week co‐culture, a collagenase solution was introduced into these chips for 2‐h incubation, followed by washing with PBS to remove collagen and residual fibroblasts. 4T1 cells captured and cultured in these chips were immunostained by Hoechst 33258 and AF488‐CK. Fluorescence images were recorded, and the number of cells were counted.

The antibody‐functionalized PDMS scaffold microchip was prepared according to previous work [39, 44, 45]. The cellulose scaffold microchip and the PDMS scaffold microchip were both introduced with the same number of MCF‐7 cells at a flow rate of 50 µL min^−1^, following by rinsing with culture medium. Next, a mixture of collagen and 10^5^ fibroblasts (using NIH/3T3 cells here) was injected into the two types of microchips to co‐culture with the captured MCF‐7 cells for 14 days. After 14‐day co‐culture, collagen was degraded, and MCF‐7 cells cultured in the two types of microchips were immunostained by Hoechst 33258 and AF488‐CK. Fluorescence images were recorded, and the number of MCF‐7 cells cultured in the chips were counted. All the above experiments were repeated at least three times, and all statistical analyses were performed at 95% confidence level. A two‐tailed *t*‐test was applied to determine the *P* values.

About 100 MCF‐7 cells were spiked into 1 mL of healthy human blood, which was then injected into an antibody‐functionalized cellulose scaffold microchip at a flow rate of 50 µL min^−1^. Following, the microchip was washing with PBS at a flow rate of 50 µL min^−1^ for 5 min to remove the non‐specifically trapped hematologic cells. Then, the collagen solution was diluted to 1 mg mL^−1^ with its pH adjusted to 7.2‐7.4, and was added with 10^5^ fibroblasts. The mixed solution was introduced into this microchip that had trapped CTCs. After solidifying the collagen at 37 C and adding culture medium, the microchip was cultured in an incubator for 7 days. The microchip, only in the presence of collagen, and the microchip with MCF‐7 cells individually cultured were taken as controls. According to the earlier described method, these microchips were immunostained by Hoechst 33258, PE‐CD45, and AF488‐CK to enumerate the number of MCF‐7 cells after capture and expansion. Moreover, after 7‐day co‐culture, the expanded MCF‐7 cells in the cellulose scaffold chip were stained by Calcein‐AM and PI for 30 min in the incubator, washed with PBS, and observed by fluorescence microscope to characterize their bioactivity.

### 4.6 Capture and Culture of CTCs by 3D Cellulose Scaffold Microchip for Tumor Xenograft Construction

The animal experiments were carried out using protocol that had undergone review and approval (approval number: HLK‐20220201‐001) by Nationalities Institutional Review Board of Wuhan Myhalic Biotechnology Co., Ltd.

First, under sterile conditions, after BalB/c mice (female, 4–6 weeks, ∼ 20 g) were anesthetized with 1% pentobarbital sodium, about 10^7^ 4T1 cells were injected into their mammary gland site in the hind limbs. These mice were continued to be raised in specific pathogen‐free (SPF) conditions until tumors grew at the injection site. When their tumors grew to 500 mm^3^, the whole blood of these mice was drawn by a sterile syringe and collected into a vacuum blood collection tube containing anticoagulant [46].

Then, the sterile antibody‐functionalized cellulose scaffold microchips were used to capture and culture of CTCs from the whole blood of these tumor‐bearing mice. The detailed operation process was the same as section 4.5. On the one hand, the microchips for capturing and culturing CTCs were stained by Hoechst 33258, PE‐CD45, and AF488‐CK to identify the number of CTCs. On the other hand, after capture and one‐month culture, collagenase solution was added to these microchips for 2‐h incubation to degrade collagen, and then these microchips were washed by culture medium to remove residual collagen and fibroblasts. Next, 0.25% trypsin solution was introduced into these microchips for about 10 min treatment, and free CTCs after culture were obtained by washing these microchips with culture medium. By microscopic manipulation, cultured CTCs with clear edges and diameters larger than 10 µm were selected and collected in the RNA lysis solution for single‐cell transcriptome sequencing and analysis. Meanwhile, tumor cells derived from primary tumor tissues of tumor‐bearing mice were used as a control.

We performed experiments to present the biocompatibility and biodegradation of the 3D cellulose scaffold in vivo. The 3D cellulose scaffold was sterilized by autoclaving. The BalB/c nude mice (female, ∼ 20 g, 6 weeks old) were always reared in SPF conditions with an environmental temperature of 22–26 C, relative humidity of 50%–60%, as well as 12‐h light and 12‐h darkness per day. The BalB/c nude mice were allowed to acclimate for 3–7 days prior to the implantation experiment. After that, three nude mice were anesthetized with 1% pentobarbital sodium by subcutaneous injection. Then, at the location of the mammary gland in the hind limbs of each nude mouse, an approximately 4‐mm incision was cut in the skin, and the sterile cell‐free cellulose scaffold was implanted into the mouse from this incision. After suturing this incision, these three nude mice were continued to be kept in SPF conditions, and the status of the mice and the degradation of the implanted scaffold were observed constantly. Six weeks later, these three nude mice were sacrificed, and their major organs (heart, liver, spleen, brain, lungs, and kidneys) were excised and fixed in paraformaldehyde for H&E staining.

Previously, the 3D cellulose scaffold microchip was applied to capture and culture CTCs from whole blood of tumor‐bearing mice. After CTCs were cultured in the microchip, the microchip was disassembled, and the cellulose scaffold with expanded CTCs was retrieved and then temporarily placed in sterile cell culture medium. Subsequently, the scaffold with expanded CTCs was surgically implanted into the mammary gland site of BalB/c nude mice. These retrieval and implantation processes were accomplished in 10 min and performed under sterile conditions to prevent contamination. Then, the state of the nude mouse and its tumor growth were observed. When its tumor reached 500 mm^3^, the tumor and its major organs were excised and fixed in paraformaldehyde for histopathological staining.

### 4.7 Constructing Patient‐Derived Xenografts by CTCs from Cancer Patient Blood

The research protocol was approved by the Institutional Review Board (IRB) of Renmin Hospital of Wuhan University (Approval No. WDRY2024‐K031), and it was conducted in line with the guidelines established by the Declaration of Helsinki. Written informed consent was obtained from all participating blood donors prior to sample collection. Fresh ethylenediamine tetraacetic acid anticoagulated blood samples were collected from three breast cancer patients. 10 mL of blood was harvested from each patient and divided into three equal volumes (about 3.3 mL of one portion). One portion of the blood was introduced into the antibody‐functionalized cellulose scaffold microchip, and CTCs were captured and enumerated, according to the steps described above. The remaining two parts of the blood were separately flowed through antibody‐functionalized cellulose scaffold microchips at a flow rate of 50 µL min^−1^. After the microchips were washed to remove hematologic cells, the mixed solution of collagen and 10^5^ fibroblasts (using HMF 7630 cells here) was injected into the microchips to co‐culture with the captured CTCs, as described previously.

Through a period of culture, one of the microchips was treated by collagenase solution, washed with PBS, as well as immunostained by Hoechst 33258, PE‐CD45, AF488‐CK, and AF647‐Ki67 to identify and enumerate the CTCs after culture. The other microchip was detached after culture, and the cellulose scaffold with expanded CTCs was obtained and implanted into the mammary gland of the BalB/c nude mouse as mentioned previously. The nude mouse was observed for tumor growth, and its body weight was recorded. And after it was sacrificed, its major organs (liver and lungs) were excised for H&E staining to characterize tumor cell metastasis. Furthermore, the immunohistochemical (IHC) staining of liver was performed using a human‐specific anti‐Ki67 monoclonal antibody (GB14102‐50, Servicebio).

### 4.8 Statistical Analysis

Statistical analyses and graph production was performed using software Origin 2019b. Experimental data were normalized prior to analysis, and processed data were presented as mean ± standard deviation (SD). The exact sample size (n), representing independent biological replicates, was provided for each dataset in the figure legends. A two‐tailed unequal variance *t*‐test was applied to assess statistically significant differences between two independent experimental groups. The threshold for statistical significance was defined as α = 0.05 for all tests, and exact *p*‐values were detailed in the results or figure legends.

## Funding

This work was supported by the National Natural Science Foundation of China (Grant nos. 21974098, 22274120, and 12175167).

## Conflicts of Interest

The authors declare no conflicts of interest.

## Supporting information




**Supporting file 1**: advs74392‐sup‐0001‐SuppMat.docx

## Data Availability

The data that support the findings of this study are available from the corresponding author upon reasonable request.
