# Supplementary material for: Capture and Culture of Circulating Tumor Cells Using a Biodegradable 3D Scaffold for Patient‐Derived Xenograft Construction
Source: Adv Sci (Weinh). 2026 Mar 17;13(23):e11148. doi: 10.1002/advs.202511148 (PMC13104101; doi:10.1002/advs.202511148)
Supplement: Supplementary file 1 — Supporting file 1: advs74392‐sup‐0001‐SuppMat.docx [file ADVS-13-e11148-s001.docx]

Supporting Information

Capture and Culture of Circulating Tumor Cells Using a Biodegradable Three-Dimensional Scaffold for Patient-Derived Xenograft Construction

Yi-Ke Wang, Ming Wang, Min Liu, Yi-Jing Chen, Li-Li Xu, Jin-Ping Zhou, Fu-Bing Wang,^*^ Shi-Bo Cheng,^*^ Min Xie,^*^ Wei-Hua Huang^*^

Y. K. Wang, Y. J. Chen, L. L. Xu, J. P. Zhou, M. Xie, W. H. Huang

College of Chemistry and Molecular Sciences, Wuhan University, Wuhan, P. R. China

E-mail: whhuang@whu.edu.cn; mxie@whu.edu.cn

S. B. Cheng

School of Laboratory Medicine, Hubei University of Chinese Medicine, Hubei Shizhen Laboratory, Wuhan, P. R. China

E-mail: shibocheng@hbucm.edu.cn

F. B. Wang

Center for Single-Cell Omics and Tumor Liquid Biopsy, Zhongnan Hospital of Wuhan University, Wuhan, P. R. China

E-mail: wangfubing@znhospital.cn

Y. K. Wang

Henan Academy of Sciences, Zhengzhou, P. R. China

M. Wang

Department of Clinical Laboratory, Renmin Hospital of Wuhan University, Wuhan, P. R. China

M. Liu

Department of Laboratory Medicine, Zhongnan Hospital of Wuhan University, Wuhan, P. R. China

**Figure S1**


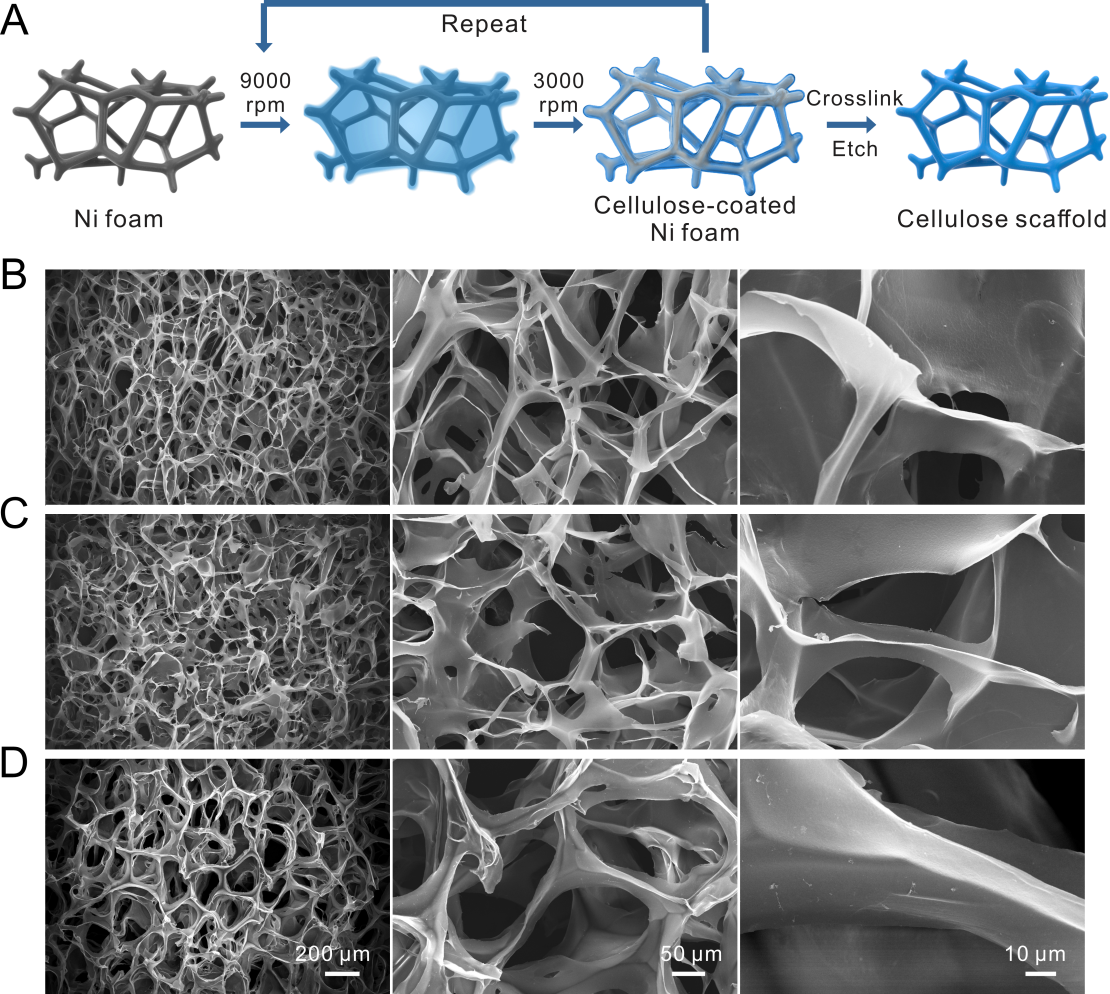


**Figure S1.** The preparation and characterization of three-dimensional (3D) cellulose scaffold. A) Diagram representing the fabrication process of 3D cellulose scaffold. B-D) Scanning electron microscopy (SEM) images of 3D cellulose scaffold fabricated from nickel (Ni) foam with 1 mm thickness at different magnifications. B): Assembling 1 layer of cellulose. C): 2 layers of cellulose. D): 3 layers of cellulose.

**Figure S2**


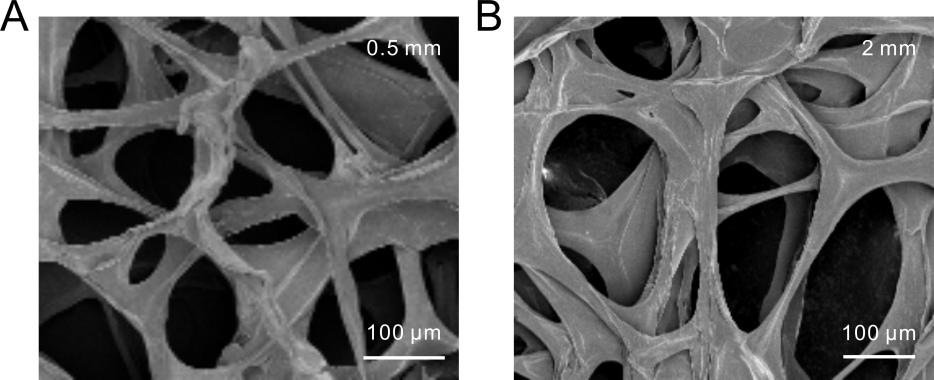


**Figure S2.** SEM images of 3D cellulose scaffolds fabricated from different Ni foams. A) Ni foam with 0.5 mm thickness. B) Ni foam with 2 mm thickness.

**Figure S3**


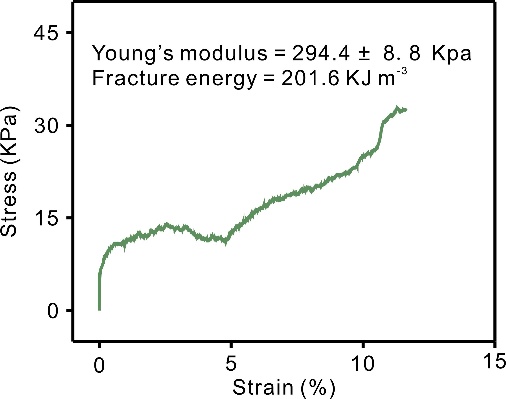


**Figure S3.** The stress-strain curve of the 3D cellulose scaffold (n = 3, the average value of Young's modulus and fracture anergy is marked in the figure.).

**Figure S4**


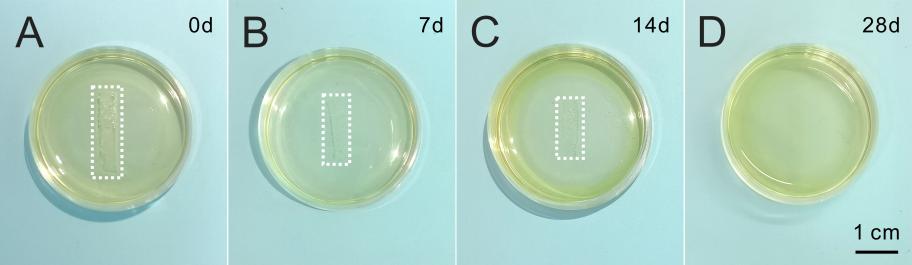


**Figure S4.** In vitro degradation of cellulose scaffold. A-D) Photos of the scaffold immersed in cellulase solution at day 0 (A), day 7 (B), day 14 (C) and day 28 (D).

**Figure S5**


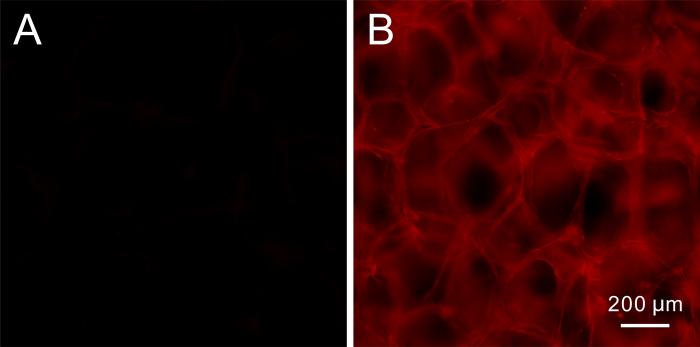


**Figure S5.** Characterization of antibody-functionalized cellulose scaffold by DyLight 594-labeled goat anti-mouse secondary antibody. A) Cellulose scaffold without modifying with antibody. B) Scaffold modified with antibody.

**Figure S6**


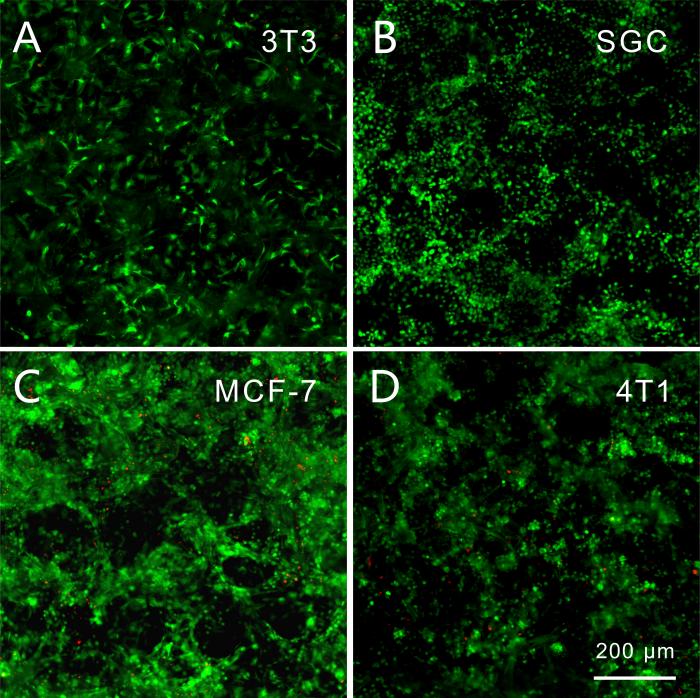


**Figure S6.** Characterizing the biocompatibility of cellulose scaffold. Fluorescence images of 3T3 cells (A), SGC cells (B), MCF-7 cells (C) and 4T1 cells (D) cultured by the cellulose scaffold for 7 days and stained by Calcein-AM (green) and propidium iodide (PI, red).

**Figure S7**


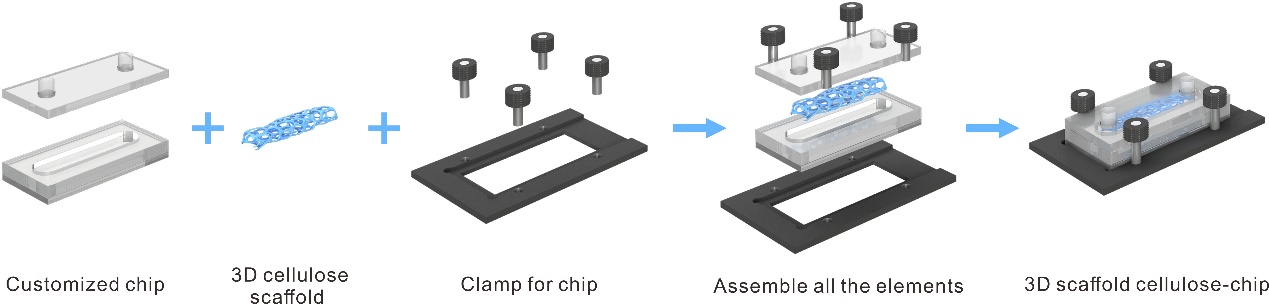


**Figure S7.** Schematic diagram representing the assembling process of 3D cellulose scaffold microchip.

**Figure S8**


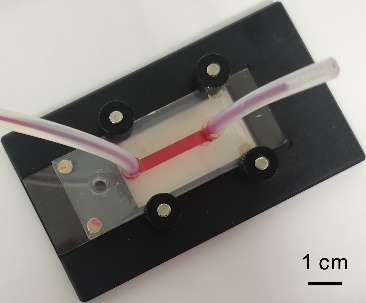


**Figure S8.** Photo of 3D cellulose scaffold microchip. The chip was introduced into the red ink.

**Figure S9**


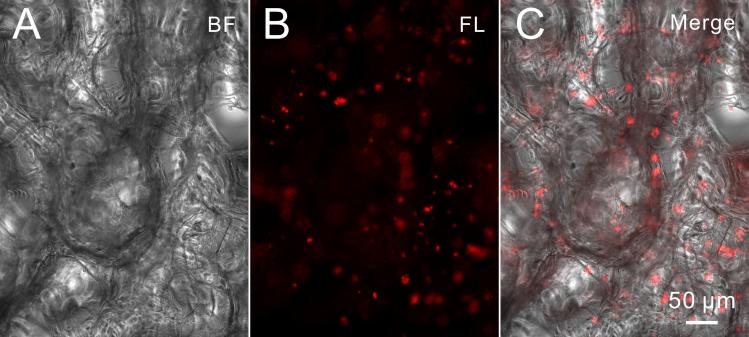


**Figure S9.** DiI-stained MCF-7 cells captured by 3D cellulose scaffold microchip. A) Bright field image of the captured MCF-7 cells in cellulose scaffold chip. B) Fluorescence image of the captured MCF-7 cells in cellulose scaffold chip. C) The merge image of bright field and fluorescence image.

**Figure S10**


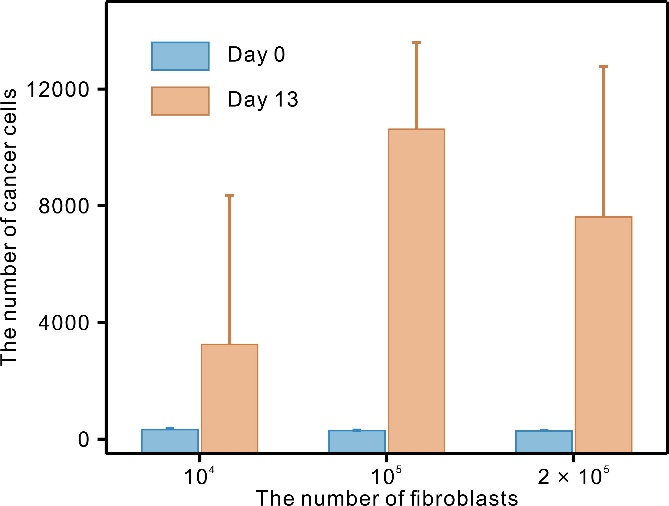


**Figure S10.** The number of 4T1 cells after capture and co-culture with different fibroblast densities in the cellulose scaffold chip (n = 3, mean ± SD).

**Figure S11**


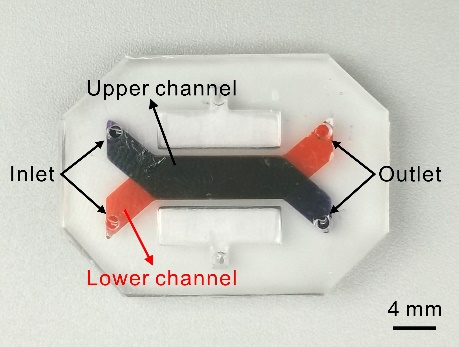


**Figure S11.** Photograph showing the dual-channel microfluidic device with two compartmentalized channels and a mid-layer thin membrane. The upper channel was introduced into black ink, while the lower channel was injected into red ink.

**Figure S12**


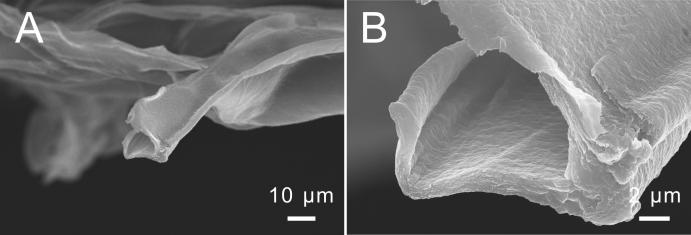


**Figure S12.** SEM images showing hollow cellulose scaffold.

**Figure S13**


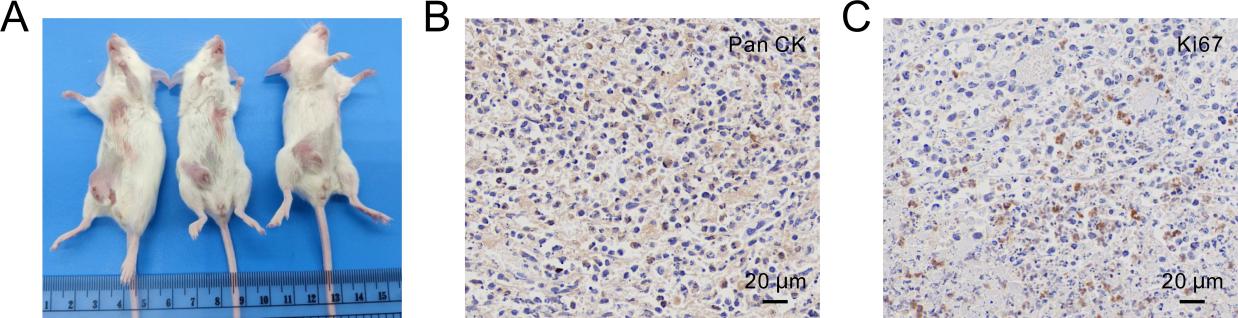


**Figure S13.** Characterization of tumor-bearing mouse models. A) Representative image of tumor-bearing BalB/c mice. B-C) Histological staining of tumor from the tumor-bearing mouse. B) Pan cytokeratin (pan-CK) staining. C) Ki67 staining.

**Figure S14**


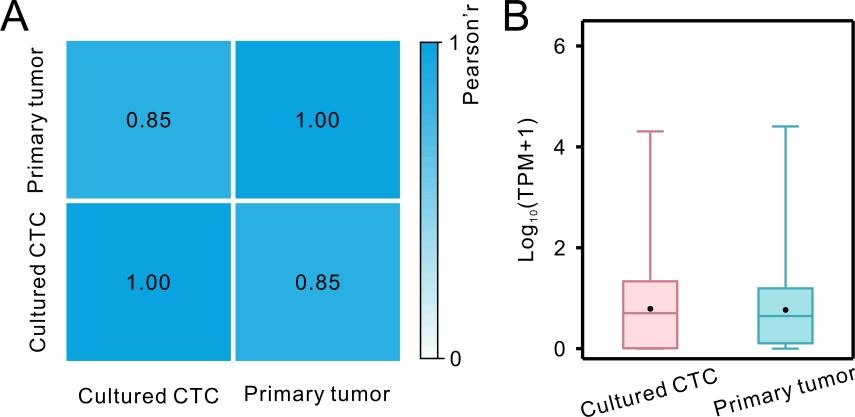


**Figure S14.** Gene expression level analysis in cultured circulating tumor cells (CTCs) and primary tumor cells. A) Heat map representing Pearson correlation coefficient between cultured CTCs by cellulose chip and primary tumor. B) Box plot of gene expression distribution in two samples.

**Figure S15**


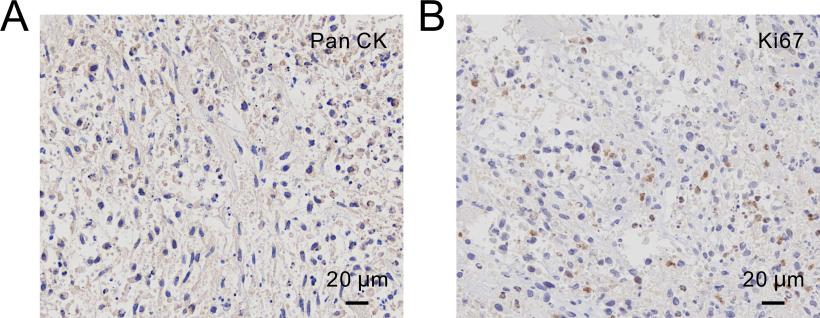


**Figure S15.** Histological staining of tumor from BalB/c nude mouse after implantation with the cellulose scaffold containing cultured CTCs. A) Pan CK staining. B) Ki67 staining.

**Figure S16**


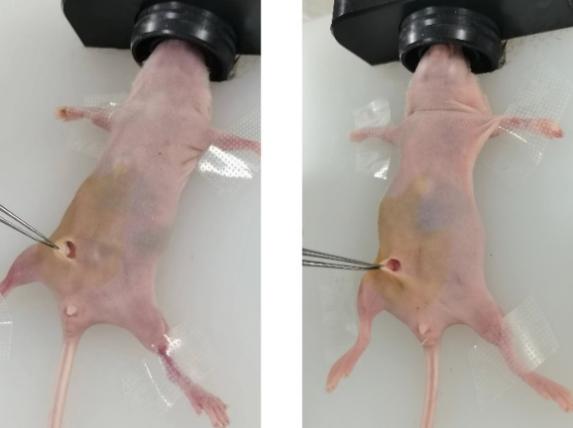


**Figure S16.** Image of BalB/c nude mouse implanting with sterile cellulose scaffold.

**Figure S17**


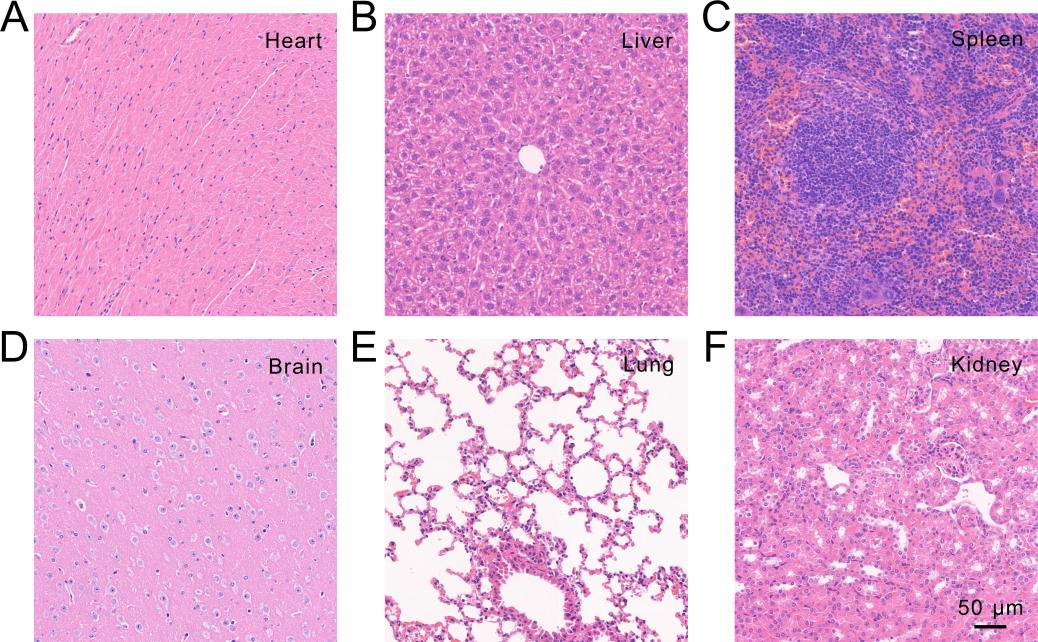


**Figure S17.** H&E staining of tissues from heart (A), liver (B), spleen (C), brain (D), lung (E), kidney (F) of BalB/c nude mouse after implanting with sterile cell-free cellulose scaffold.

**Figure S18**


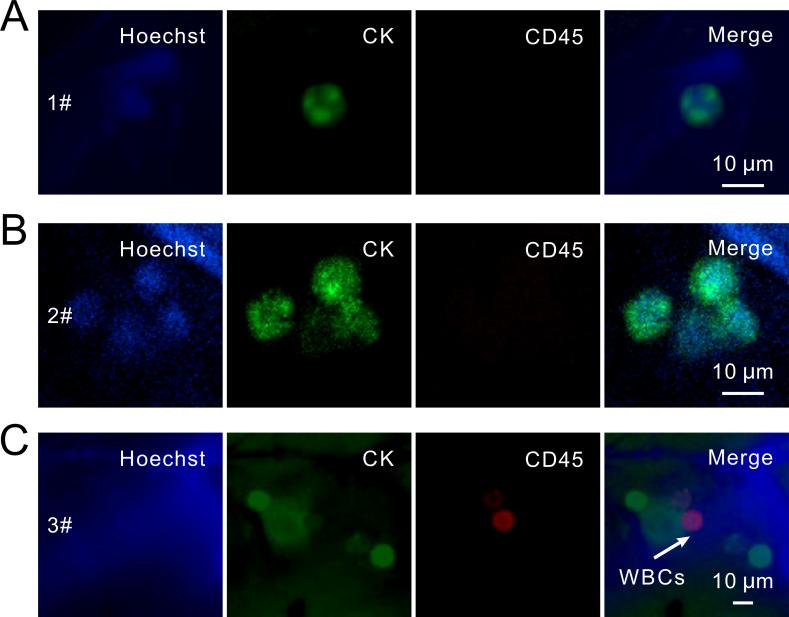


**Figure S18.** Representative fluorescence images of CTCs captured from breast cancer patient. A) Patient 1#. B) Patient 2#. C) Patient 3#.

**Figure S19**


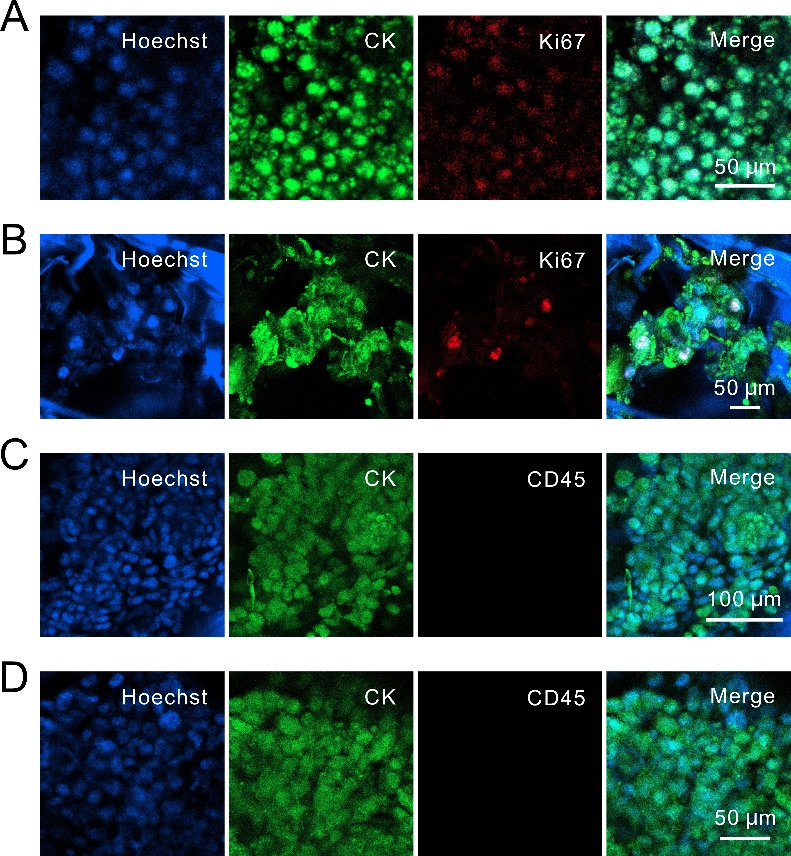


**Figure S19.** Representative fluorescence images of expanded CTCs after capture and co-culture in the cellulose chip.

**Figure S20**


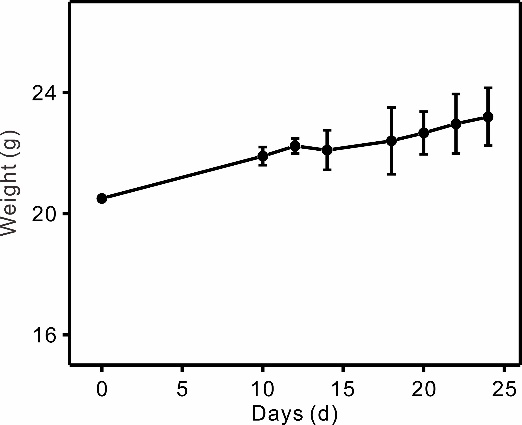


**Figure S20.** The curve showing changes in body weight of mice with implantation time (n = 18, mean ± SD).

**Figure S21**


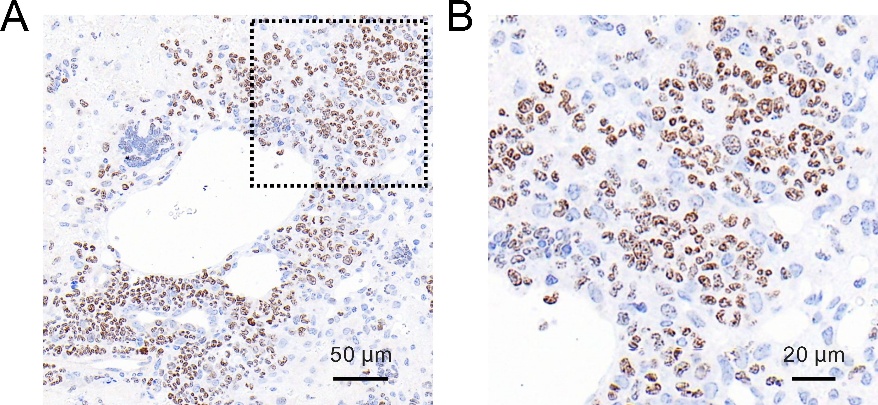


**Figure S21.** (A) Histological staining for Ki67 of liver tissue from a Balb/c nude mouse implanted with the cellulose scaffold containing cultured CTCs from breast cancer patient 2#. (B) A higher-magnification view of the area outlined by the dashed rectangle in (A).

**Table S1.** Quantitative data of CTCs isolated from cancer patient blood samples by 3D cellulose scaffold microchip.

| **Samples**  **No.** | **Cancer type** | **Gender** | **Age** | **Captured CTCs (per mL)** |
| --- | --- | --- | --- | --- |
| 1 | Lung cancer | M | 53 | 6 |
| 2 | Lung cancer | F | 51 | 5 |
| 3 | Lung cancer | M | 53 | 8 |
| 4 | Lung cancer | M | 60 | 10+1 cluster |
| 5 | Lung cancer | M | 72 | 3 |
| 6 | Lung cancer | F | 46 | 19 |
| 7 | Lung cancer | F | 62 | 11 |
| 8 | Lung cancer | M | 63 | 12 |
| 9 | Nasopharyngeal cancer | M | 30 | 4 |
| 10 | Esophageal cancer | F | 52 | 18+2 clusters |
| 11 | Esophageal cancer | F | 69 | 15+1 cluster |
| 12 | Gastric cancer | F | 49 | 5+1 cluster |
| 13 | Gastric cancer | F | 61 | 15+2 clusters |
| 14 | Gastric cancer | M | 53 | 36+3 clusters |
| 15 | Gastric cancer | F | 65 | 5+1 cluster |

**Table S2.** Quantitative data of the isolated CTCs and the expanded CTCs from three breast cancer patient blood samples by 3D cellulose scaffold microchip.

| **Samples**  **No.** | **Captured CTCs** | **expanded CTCs** | **Amplification fold** |
| --- | --- | --- | --- |
| 1 | 9 | 38 | 4.2 |
| 2 | 35 | 648 | 18.5 |
| 3 | 12 | 35 | 2.9 |
